# Dysregulated RNA editing of *EIF2AK2* in polycystic ovary syndrome: clinical relevance and functional implications

**DOI:** 10.1186/s12916-024-03434-8

**Published:** 2024-06-10

**Authors:** Fan-Sheng Kong, Junjie Feng, Jin-Ping Yao, Yinghua Lu, Tao Guo, Meng Sun, Chun-Yan Ren, Yun-Yun Jin, Yaping Ma, Jian-Huan Chen

**Affiliations:** 1 Laboratory of Genomic and Precision Medicine, Wuxi School of Medicine, Jiangnan University, Wuxi, Jiangsu, China; 2 Department of Pediatrics, Affiliated Hospital of Jiangnan University, Wuxi, 214122 Jiangsu China; 3 Department of Reproductive Medicine, Affiliated Hospital of Jiangnan University, Wuxi, 214122 Jiangsu China; 4 Joint Primate Research Center for Chronic Diseases, Institute of Zoology of Guangdong Academy of Science, Jiangnan University, Wuxi, Jiangsu, China; 5 Jiangnan University Brain Institute, Wuxi, Jiangsu China

**Keywords:** Polycystic ovary syndrome, RNA editing, *EIF2AK2*, *ADAR*, Clinical feature, Peripheral blood, Dehydroepiandrosterone, MAPK pathway

## Abstract

**Background:**

Polycystic ovary syndrome (PCOS) is a prevalent endocrine disorder affecting women of reproductive ages. Our previous study has implicated a possible link between RNA editing and PCOS, yet the actual role of RNA editing, its association with clinical features, and the underlying mechanisms remain unclear.

**Methods:**

Ten RNA-Seq datasets containing 269 samples of multiple tissue types, including granulosa cells, T helper cells, placenta, oocyte, endometrial stromal cells, endometrium, and adipose tissues, were retrieved from public databases. Peripheral blood samples were collected from twelve PCOS and ten controls and subjected to RNA-Seq. Transcriptome-wide RNA-Seq data analysis was conducted to identify differential RNA editing (DRE) between PCOS and controls. The functional significance of DRE was evaluated by luciferase reporter assays and overexpression in human HEK293T cells. Dehydroepiandrosterone and lipopolysaccharide were used to stimulate human KGN granulosa cells to evaluate gene expression.

**Results:**

RNA editing dysregulations across multiple tissues were found to be associated with PCOS in public datasets. Peripheral blood transcriptome analysis revealed 798 DRE events associated with PCOS. Through weighted gene co-expression network analysis, our results revealed a set of hub DRE events in PCOS blood. A DRE event in the eukaryotic translation initiation factor 2-alpha kinase 2 (*EIF2AK2*:chr2:37,100,559) was associated with PCOS clinical features such as luteinizing hormone (LH) and the ratio of LH over follicle-stimulating hormone. Luciferase assays, overexpression, and knockout of RNA editing enzyme adenosine deaminase RNA specific (*ADAR*) showed that the ADAR-mediated editing cis-regulated *EIF2AK2* expression. *EIAF2AK2* showed a higher expression after dehydroepiandrosterone and lipopolysaccharide stimulation, triggering changes in the downstrean MAPK pathway.

**Conclusions:**

Our study presented the first evidence of cross-tissue RNA editing dysregulation in PCOS and its clinical associations. The dysregulation of RNA editing mediated by ADAR and the disrupted target *EIF2AK2* may contribute to PCOS development via the MPAK pathway, underlining such epigenetic mechanisms in the disease.

**Supplementary Information:**

The online version contains supplementary material available at 10.1186/s12916-024-03434-8.

## Background

Polycystic ovary syndrome (PCOS) is a common endocrine disorder affecting reproductive-aged women, characterized by hyperandrogenism, ovulatory dysfunction, and polycystic ovaries [1, 2]. PCOS is associated with several long-term complications, including atherogenic dyslipidemia, non-alcoholic fatty liver disease, systemic inflammation, type 2 diabetes, and mental health disorders [3–6]. Despite its prevalence and impact on women's health, the etiology of PCOS remains poorly understood. Multiple factors, such as genetics, insulin resistance, and chronic inflammation, contribute to the development of PCOS as a complex disorder [7–10].

Epigenetics is important in understanding gene-environment interaction [11, 12]. Epigenetic changes play significant roles in PCOS pathogenesis [13, 14]. Studies suggest that PCOS is associated with changes in DNA methylation patterns and implicate how DNA methylation changes could influence ovarian function and insulin resistance in PCOS [15, 16]. Additionally, histone modifications and non-coding RNAs could regulate gene expression and contribute to the complex pathophysiology of PCOS [17–19]. In addition, epigenetic changes could be involved in reprogramming immune cells, which might contribute to the inflammatory and metabolic features of PCOS [20]. Moreover, targeting DNA methylation epigenetics has been shown to be a novel, promising therapeutic strategy for PCOS [21]. In spite of such advances in PCOS epigenetics, RNA editing, a post-transcriptional epigenetic process that alters RNA sequences by inserting, deleting, or substituting nucleotides, remains to be further elucidated in PCOS. RNA editing could be involved in various biological and pathological processes, such as gene expression regulation, RNA splicing, and inflammation [22–26]. Dysregulation of RNA editing has been associated with several diseases, such as inflammatory disorders, cardiovascular and neuropsychiatric disorders, and cancer [27–30]. Adenosine-to-inosine (A-to-I) RNA editing, catalyzed by the adenosine deaminase acting on the RNA (*ADAR*) family, is the most common form of RNA editing in mammals [31]. Our recent study identified potential associations of RNA editing in granulosa cells with PCOS [32]. However, the actual role of RNA editing, especially its association with clinical features and the underlying mechanisms in PCOS, remains unclear.

To explore the potential multiple tissue involvement of A-to-I RNA editing in PCOS, we conducted a comprehensive search for publicly available PCOS RNA-Seq datasets. We also performed transcriptome sequencing on peripheral blood samples collected from both PCOS patients and healthy controls. By identifying dysregulated RNA editing, especially that associated with innate immune response in PCOS from these RNA-Seq data, we further conducted functional validation to assess the biological significance of RNA editing in the eukaryotic translation initiation factor 2-alpha kinase 2 (*EIF2AK2*) gene, which encodes a key protein kinase in the innate immune response and could potentially contribute to the inflammatory feature of PCOS [33]. Our findings provide new insights into the molecular mechanisms underlying the pathogenesis and epigenetically dysregulated immune response of PCOS, which could be important for developing innovative diagnostic and therapeutic approaches for the prevalent endocrine disorder in women.

## Methods

### Dataset search and download

Ten raw RNA sequencing datasets of PCOS cohorts were downloaded from the European Nucleotide Archive (ENA) and the National Center for Biotechnology Information (NCBI) Sequence Read Archive (SRA) databases (Table 1). Specifically, PRJNA576231, PRJNA649934, PRJNA679416, PRJNA707301, and PRJNA794860 contain a total of thirty-six granulosa cell samples from eighteen PCOS and eighteen controls. PRJNA649934 also contains twelve oocyte samples. PRJNA540679 contains eleven T helper cell samples. PRJNA645705 contains eight placenta samples. PRJNA719824 and PRJNA938949 contain 180 endometrial stromal cells and six endometrium samples, respectively. PRJNA798018 contained eight abdominal adipose tissue samples and eight gluteofemoral adipose tissue samples. Dataset PRJNA386593, which contains wild-type (WT) and *ADAR* knockout (ADAR_KO) cell samples, was used to analyze the RNA editing level after *ADAR* knockout [34]. Besides, to analyze the expression level of *Eif2ak2* in the mouse model, dataset PRJNA659669, which includes ovarian samples from control (CON) and prenatally androgenized mice, was also downloaded from NCBI [35]. PRJNA993124 was used to validate the effect of lipopolysaccharide (LPS) treatment, which contains THP1 cell samples treated with LPS and PBS, respectively. PRJNA554006 contains *EIF2AK2* overexpression and control HeLa cell samples, and PRJNA850448 contains control (CON) and *EIF2AK2 *knockout A549 lung cancer cell samples.

**Table 1:** Details of the public datasets of PCOS cohorts included in the current study

No	Study Bioproject ID	Author, year	Country^a^	Sample size	Sample type	Sequencing platform
1	PRJNA540679	Stepto NK, 2019 [20]	Denmark	Con:7, PCOS:4	T helper cells	Illumina NextSeq 500
2	PRJNA576231	Kang Y, 2021 [36]	China	Con:3, PCOS:3	Granulosa cells	Illumina HiSeq 2000
3	PRJNA645705	Qu F,2020 [37]	China	Con:3, PCOS:5	Placenta	Illumina HiSeq 2500
4	PRJNA649934	Liu L, 2021 [38]^b^	China	Con:4, PCOS:4	Granulosa cells	HiSeq X Ten
5	PRJNA649934	Liu L, 2021 [38]^b^	China	Con:6, PCOS:6	Oocyte	HiSeq X Ten
6	PRJNA679416	Huang D, 2021 [39]	America	Con:3, PCOS:3	Granulosa cells	Illumina NovaSeq 6000
7	PRJNA707301	Zhao H, 2021 [40]	China	Con:5, PCOS:5	Granulosa cells	Illumina HiSeq 2500
8	PRJNA719824	Piltonen TT, 2021 [41]	Estonia	Con:96, PCOS:84	Endometrial stromal cells	Illumina NextSeq 500
9	PRJNA794860	Pang Y, 2022 [42]	China	Con:3, PCOS:3	Granulosa cells	Illumina NovaSeq 6000
10	PRJNA938949	Elin Org, 2021[43]	China	Con:3, PCOS:3	Endometrium	Illumina HiSeq 2500
11	PRJNA798018	Smith SR, 2022 [13]^b^	America	Con:4, PCOS:4	Abdominal adipose tissues	Illumina HiSeq 2500
12	PRJNA798018	Smith SR, 2022 [13]^b^	America	Con:4, PCOS:4	Gluteofemoral adipose tissues	Illumina HiSeq 2500

Publicly available RNA-Seq datasets from ten PCOS studies are found and acquired for the current study

^a^Study country: The country of the study cohort that met the inclusion criteria is included and reanalyzed in our study

^b^Datasets PRJNA649934 and PRJNA798018 contain samples from two distinct tissue types and are analyzed for each tissue type separately

### Peripheral blood sample collection

This study was approved by the Ethics Committee of Jiangnan University (JNU20230611RB18) and was conducted according to the principles outlined in the Declaration of Helsinki. All participants were at least 18 years old and had their written informed consent collected after explaining the details of the study. The study enrolled a total of 28 participants with or without PCOS. The discovery cohort comprised ten controls without PCOS and twelve naïve patients with PCOS. The validation cohort consisted of three controls without PCOS and three naïve patients with PCOS. The inclusion criteria for the PCOS patients were based on the Rotterdam Consensus (2003), which required the presence of at least two of the following criteria: ovulatory dysfunction, polycystic ovarian morphology, and clinical and/or biochemical hyperandrogenism.

Participants were excluded if they met any of the following criteria: (1) use of hormonal contraceptives or antidiabetic medication within three months before the study; (2) history of gastrointestinal disease, active infections, smoking, or a body mass index below 18 kg/m^2^; (3) adrenal disorders; (4) currently pregnant; (5) Cushing’s syndrome; or (6) presence of chronic diseases such as chronic kidney or liver diseases.

Peripheral blood samples from the participants were collected from the basilic vein using EDTA-containing vacutainer tubes. The samples were kept on ice before RNA extraction within 24 h.

### Blood hormone testing

Serum levels of reproductive hormones, including follicle-stimulating hormone (FSH), luteinizing hormone (LH), LH/FSH ratio, testosterone (T), dehydroepiandrosterone sulfate (DHEA-S), and estradiol, were measured using an immunochemiluminometric assay (ICMA, DxI800, BECKMAN).

### RNA extraction and transcriptome sequencing

RNA was extracted from the blood samples using the TIANGEN RNA simple Total RNA kit (DP419, China), and RNA concentration was measured using Qubit 3.0. RNA quality was evaluated using the 2100 BioAnalyzer (Agilent Technologies). Samples with RIN scores of ≥ 6.0 (indicating good RNA integrity) were used for library construction using the polyA method and the libraries were sequenced using the Illumina NovaSeq 6000 platform.

### RNA editing analysis

As described in our previous study, after downloading raw data, FASTQC was used to assess the quality of raw data [44]. RNA STAR (Version 2.7.0e) was used to align and map the RNA-seq reads to the reference genome, which generated alignment files in BAM format [45]. To ensure the quality of the alignment results, the BAM files were subsequently filtered using SamTools (Version 1.9) to remove optical duplications and retain only reads that were uniquely mapped to the genome [46]. Additionally, to further improve the accuracy of the base quality scores and reduce potential sequencing errors, the BAM files were subjected to base quality score recalibration using GATK (Version 4.1.3) [47].

To identify RNA single-nucleotide variations (SNVs) in our study, VarScan (Version 2.4.4) was used to call and analyze the SNVs. The variant calling criteria were set as follows: a minimum base quality of 25, a minimum sequencing depth of ten, a minimum alternative allele depth of two, and an alternative allele frequency (AAF) of 1% or higher. The fpfilter command in VarScan was used with its default parameters to filter these variants to ensure the accuracy of variant calling. SNVs were further annotated using the Ensembl Variant Effect Predictor (VEP) to gain insight into their functional significance [48, 49]. Inosine was detected as guanine in RNA-Seq data, and only A-to-G SNVs on the coding strand and T-to-C SNVs on the non-coding strand were included in subsequent analysis. SNVs that met the following criteria were removed unless they were annotated as RNA editing events in the REDIportal V2.0 database: (1) located in homopolymer runs of five nucleotides (nt) or more, simple repeats, or the mitochondria; (2) within six nt from splice junctions, one nt from insertions or deletions, or 4% to the ends of reads; (3) annotated in the dbSNP database Build 142; (4) with an AAF of 100% or between 40 and 60% in more than 90% of the analyzed samples [44, 50, 51]. We further focused on high-confidence A-to-I RNA editing events with editing levels ≥ 1% observed in at least two samples or annotated in the REDIportal V2.0 database [50].

### Gene expression quantification of RNA-Seq data

Rsubread (Version 2.14.2) was used to calculate the pseudo-counts of RNA expression from the BAM files. Subsequently, edgeR (Version 3.7) was employed to calculate the transcripts read per thousand bases per million mappings (TPM) for gene expression [52, 53].

### Identification of hub differential editing events

Since most differential editing events have unknown effects, it was important to identify hub differential editing events to gain insights into the underlying molecular mechanisms and potential functional implications in the context of the study. In brief, the R package WGCNA was used to construct the WGCNA network [54]. The function of “pickSoftThreshold” was used to assess the value of powers in generating co-expression networks. The STRING database (http://string-db.org) was used to construct the protein-protein interactive (PPI) network with an interaction score cutoff > 0.4.

Then the top ten differential editing events in the major functional module were extracted by the degree centrality algorithm in the software Cytoscape (Version 3.9.1).

Moreover, random forest analysis and gene set enrichment analysis (GSEA) were performed using the OECloud tools (https://cloud.oebiotech.cn).

### Gene function enrichment analysis

To gain insight into the biological functions of the edited genes, we performed Gene Ontology (GO) and Kyoto Encyclopedia of Genes and Genomes (KEGG) pathway analyses using Enrichr [55]. A significance cutoff of *P* < 0.05 was applied to identify enriched GO terms and KEGG pathways.

### RNA-binding protein binding site prediction

The RBPmap (http://rbpmap.technion.ac.il) web tool was used to predict potential RNA-binding protein (RBP) binding sites associated with these editing events [56]. Wordcloud package (Version 2.6) was used to visualize the predicted RBPs.

### Cell culture, transfection, quantitative PCR, and cell viability assays

Luciferase reporter plasmids containing wild-type (WT) and edited-type (ET) variants of *EIF2AK2* 3′-UTR were constructed using the psiCHECK2-Modified plasmid. Human embryonic kidney (HEK293T) cells were cultured in 24-well plates and transfected with the reporter plasmids using Lipofectamine 3000 (Thermo Fisher Scientific, Waltham, MA). After 48 h of transfection, the activities of both firefly luciferase (LUC) and Renilla luciferase (RLUC) were measured with a Dual-Luciferase Reporter System Kit (Cat# DL101-01, Vazyme, China).

Expression plasmids containing the ADAR-p110 and ADAR-p150 isoforms were purchased from GenePharma (China). The two plasmids were transfected into HEK293T cells using Lipofectamine 3000. The cells were collected, and total RNA was extracted from the samples 48 h after transfection.

The real-time quantitative PCR were performed using SYBR Green (Nanjing Vazyme Biotech Co., Ltd) on the LightCycler® 480 II Real-time PCR System (Roche). The 2^(-ΔΔCt) method was used to determine the relative expression levels of *EIF2AK2* and *ADAR* mRNA. The housekeeping gene beta-actin (*ACTB*) was used as an internal control for expression normalization. Each sample was repeated at least three times.

To investigate the correlation between dehydroepiandrosterone (DHEA) and the site *EIF2AK2*:chr2:37,100,559 in ovarian function, human granulosa (KGN) cells were stimulated with DHEA at varying concentrations (0, 10^−8^, 10^−7^, 10^−6^,10^−5^, 10^−4^ mol/L) for 72 h [57, 58]. To explore the effect of LPS, 1 µg/mL LPS (L8880, Solarbio, China) was used to treat KGN cells for 24 h [59]. Subsequently, Sanger sequencing and real-time quantitative PCR were used to measure RNA editing and gene expression levels, respectively.

Cell viability was evaluated using the CCK-8 Cell Counting Kit assay (C0042, Beyotime, China) according to the manufacturer’s guidelines. In brief, cells were seeded into 96-well plates at an initial density of 2 × 10^3^ cells per well. Subsequently, the CCK-8 solution was applied to each well at specified time points and incubated for 2 h at 37 °C. Following incubation, the fluorescence intensity at 450 nm was quantified using a microplate reader (Synergy H1, BioTek).

### Statistical analysis

To identify potential differential RNA editing, the generalized linear model (GLM) method and likelihood ratio test (LRT) were used to compare RNA editing levels between PCOS and controls. Additionally, intergroup gene expression levels were compared using the Student's *t*-test. A cutoff of *P* < 0.05 was applied to determine statistical significance. The *Pearson* correlation method was used to assess the correlation between RNA editing and gene expression, and calculate the correlation coefficient (*r*) and corresponding *P*-value. Principal component analysis (PCA) of A-to-I RNA editing was performed using the prcomp function in R (Version 3.6.3) and visualized using the ggplot2 package (Version 2.2.1). Receiver operating characteristic (ROC) curve analysis was conducted using R.

## Results

### Dysregulated A-to-I RNA editing in PCOS across different tissue types in public datasets

**Fig. 1:**
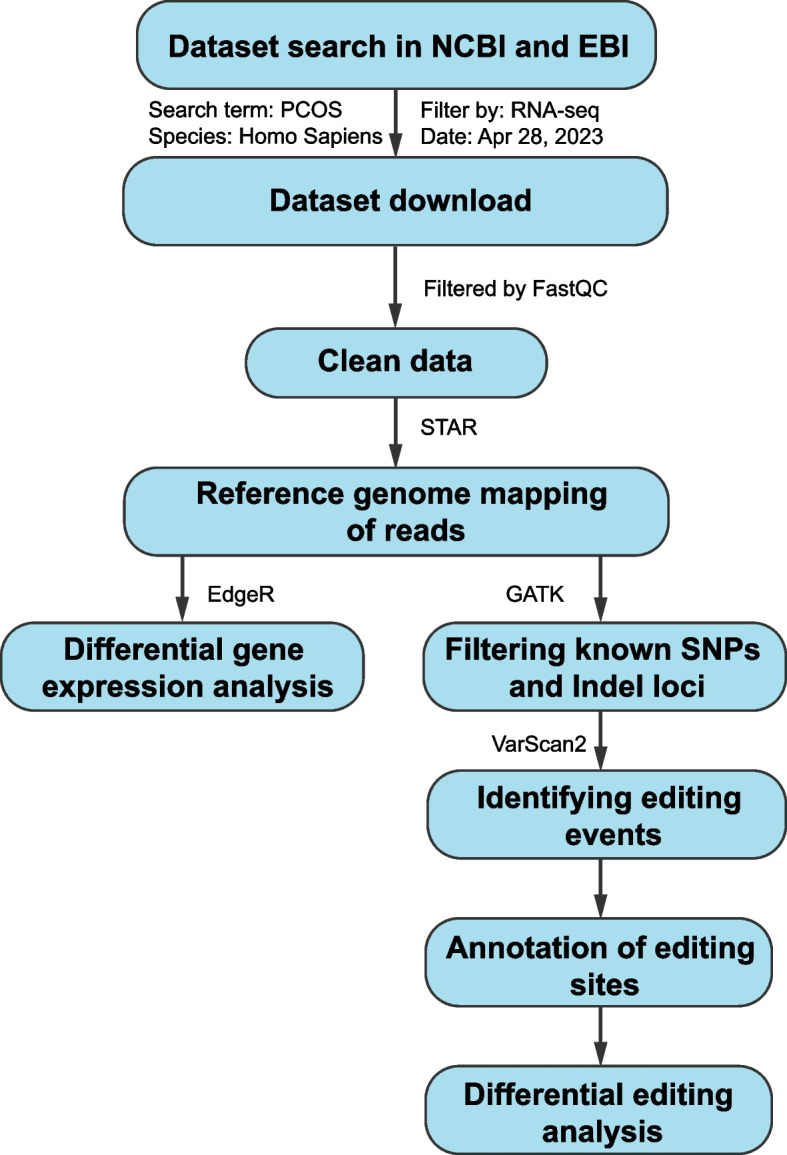
A scheme of the dataset curation and analysis procedure. The flowchart includes the initial collection of RNA-Seq data from databanks, followed by data cleaning, mapping to reference genomes, and identifying RNA editing events. Details of the analysis procedure are given in the methods

**Fig. 2:**
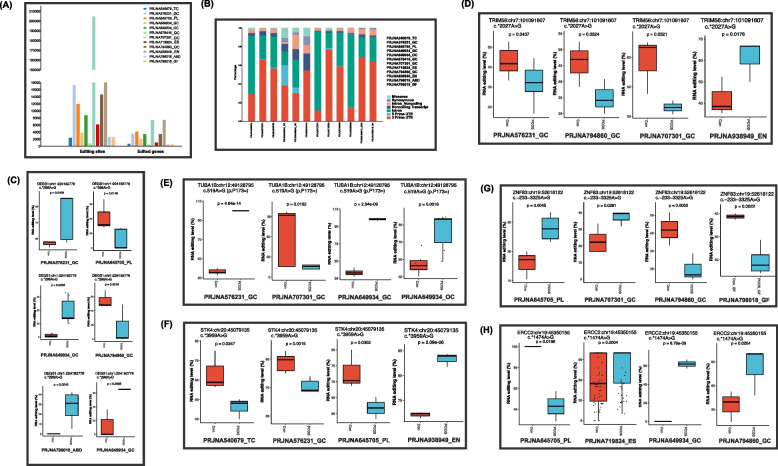
A-to-I RNA editing profiles of multiple tissues identified by epitranscriptomic analysis of publicly available PCOS cohorts. **A** The number of edited genes and A-to-I RNA editing events in each dataset. **B** The distribution of A-to-I RNA editing events across different tissue types according to their variant functional categories, according to annotation by VEP. **C** UpSet plot showing overlapped differential A-to-I RNA editing events among datasets. **D**–**I** Common differential editing events shared by at least three datasets. Differential A-to-I RNA editing events are identified by comparing the editing level (G/the sum of A and G) between PCOS and controls using the GLM method and defined as those with GLM *P* < 0.05. GLM, generalized linear model

Figure 1 shows an overview of the dataset curation and analysis procedure, including RNA sequencing, alignment, mapping, and identification of A-to-I RNA editing recognized as A-to-G SNVs in RNA-Seq, with the corresponding genomic location and the editing level (the percentage of G over the sum of A and G) of each editing events. Using such a standard procedure PCOS RNA-Seq data from multiple datasets was analyzed to identify A-to-I RNA editing across different tissue types (Fig. 2A, Additional file 1: Table S1). Notable variations in the number of A-to-I RNA editing events were observed among different tissues and datasets, pointing to the potential heterogeneity of RNA editing and the importance of cross-tissue cohort and tissue analysis in studies on RNA editing.

To investigate the dysregulated RNA editing across different tissues in PCOS, we then used the GLM method to identify differential RNA editing (DRE) by comparing the RNA editing level between PCOS and controls. We first looked into the distribution of these DRE events according to their variant functional categories. Most of these DRE events were located in the 3′-untranslated regions (3′-UTR) and intronic regions across all tissues (Fig. 2B, Additional file 1: Table S2), which might be involved in gene expression regulation.

In addition, Additional file 2: Fig S1 shows the PCA results of the DRE events in various datasets/tissues, in which the samples of PCOS and controls were well separated, suggesting that the DRE could have a considerable contribution to the difference between PCOS and controls.

Although most of these DRE events were specific to each tissue type, we then focused on the DRE and edited genes shared among public datasets (Additional file 2: Table S3, Additional file 3: Fig S2A). Our analysis identified 84 key DRE events defined to be observed in at least three datasets. Notably, the top six key DRE events were shown in Fig. 2C–H, which were consistently identified in four or more datasets, including *DEGS1*:chr1:224,192,776, *TUBA1B*:chr12:49,128,795, *TRIM56*:chr7:101,091,607, *STK4*:chr20:45,079,135, *ERCC2*:chr19:45,350,155, and *ZNF83*:chr19:52,618,122. These key DRE events thus highlighted common substantial changes across multiple tissues in PCOS.

Granulosa cells, crucial for follicular development and ovulation, are intimately linked to the anovulation and metabolic abnormalities observed in PCOS [60–62]. To better understand the potential role of A-to-I RNA editing in granulosa cells, we also conducted a pairwise comparison and identified consistent DRE in granulosa cells across the five datasets (Table 2). These findings highlight the importance of cross-population validation in investigating PCOS-associated RNA editing.

**Table 2:** Consistent DRE events in granulosa cells in different datasets

Pairwise comparison among datasets	Number of consistent common DRE events	Shared differentially edited genes
PRJNA576231 vs PRJNA649934_GC	9	*TUBA1B*,* PRKX*,* RBM3*,* MAGT1*,* MAVS*,* DEGS1*,* PSMB2*,* PLBD2*,* LDHAP4*
PRJNA57623 vs PRJNA707301	21	*AASS*,* AC005261.1*,* AC013394.1*,* AC087721.2*,* ACTR3*,* ADAMTS9*,* ANAPC16*,* AP3S2*,* CPT1A*,* CYP20A1*,* FAM126B*,* KAT8*,* NOP14*,* PAICS*,* PRKX*,* SCARB1*,* SMIM14*,* STK4*,* TRIM56*,* VHL*
PRJNA576231 vs PRJNA794860	30	*AC018638.5*,* APOBEC3C*,* CFLAR*,* CPM*,* CRCP*,* GNL3L*,* GNPNAT1*,* H2AZ2*,* HILPDA*,* HOOK3*,* LONP2*,* MAVS*,* METTL7A*,* NEBL*,* NUP43*,* OPA3*,* PHLDA1*,* PRKAR2A*,* PSMB2*,* PTPN14*,* RBBP4*,* RBM3*,* SCARB1*,* SLC25A3*,* SLC26A2*,* SNRPD3*,* TRIM56*,* UBE2G2*
PRJNA707301 vs. PRJNA649934_GC	1	*SIGLEC11*
PRJNA794860 vs PRJNA649934_GC	11	*ERCC2*,* CMC2*,* HILPDA*,* USP14*,* RBM3*,* GATC*,* SLC44A3-AS1*,* GINM1*,* NBPF14*,* FLNB*
PRJNA679416 vs PRJNA794860	1	*EIF3I*
PRJNA707301 vs PRJNA794860	19	*C005261.1*,* DFFA*,* EMP2*,* H6PD*,* MAVS*,* MDM4*,* NFATC2IP*,* NUP155*,* PAICS*,* PHAX*,* PPP1R12B*,* RBM3*,* RPL30*,* SCARB1*,* SLC44A3-AS1*,* STX2*,* TRIM56*

To provide possible functional implications, we employed the RBPmap tool to predict RBPs that could bind to the key DRE sites shared by multiple tissues/datasets. Our analysis revealed several RBPs, including RNA-binding motif protein 45 (RBM45), RNA-binding motif single-stranded interacting protein 3 (RBMS3), Y-box binding protein 1 (YBX1), and DAZ-associated protein 1 (DAZAP1) as the most frequent RBPs (Binding sites frequencies > 8) that bound to the DRE events (Additional file 4: Fig S3). These RBPs are likely crucial for the differential RNA editing observed across tissues, indicating their potential roles in the pathogenesis of PCOS.

### Differentially edited genes in public PCOS RNA-Seq datasets

We compared the sets of differentially edited genes (DEGs) in each dataset to identify the common DEGs across multiple tissues/studies. 480 DEGs that showed differentially edited in at least three datasets were recognized as common DEGs. The MDM2 proto-oncogene (*MDM2*) gene was among the most frequent DEGs that were found in eleven out of the twelve datasets (Additional file 2: Table S4, Additional file 3: Fig S2B). In addition, we found that several other genes were also consistently differentially edited across multiple datasets (at least eight datasets), such as small integral membrane protein 14 (*SMIM14*), anaphase-promoting complex subunit 16 (*ANAPC16*), mitochondrial antiviral-signaling protein (*MAVS*), and aldehyde dehydrogenase 6 family member A1 (*ALDH6A1*).

**Fig. 3:**
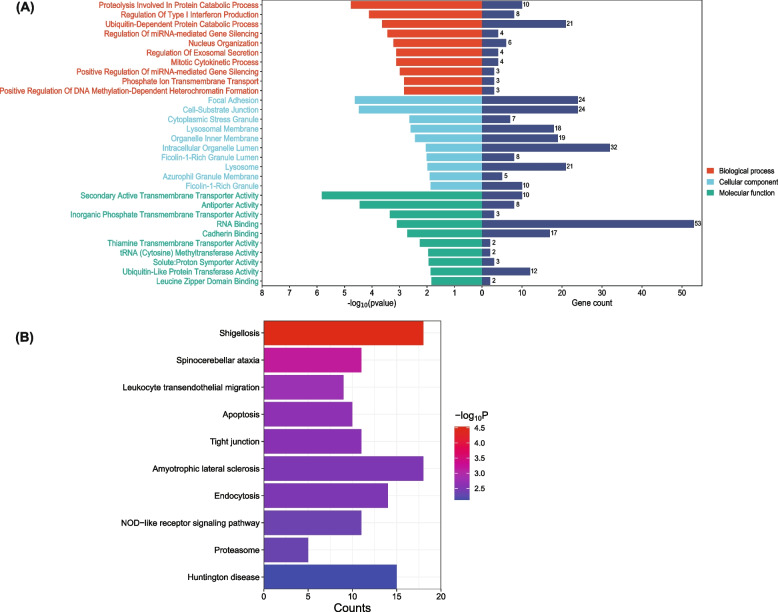
Differentially edited genes between PCOS and controls across multiple tissues/cohorts. **A** UpSet plot showing overlapped differentially edited genes in three or more datasets. GO (**B**) and KEGG pathway enrichment analysis (**C**) on differentially edited genes shared by three or more datasets. Differential editing genes are defined to contain at least one differential RNA editing event between PCOS and controls analyzed using the GLM method *P* < 0.05. GLM, generalized linear model

We then conducted an enrichment analysis to explore the potential function of these 480 common DEGs. Our results revealed these genes were primarily involved in the cellular response to Apoptosis, NOD − like receptor signaling pathway, RNA Binding, Lysosome, and Ubiquitin-Dependent Protein Catabolic Process (Fig. 3A, B). Additionally, genes involved in ubiquitin-mediated proteolysis and Endocytosis (Additional file 2: Table S5–8), such as *MDM2*, could play a potential role in PCOS.

### Clinical characteristics of the PCOS cohort with blood samples

The clinical characteristics of twelve women with PCOS and ten age-matched control women without PCOS, who had their blood samples collected, was shown in Table 3. The average testosterone (T) level in controls and PCOS was 0.76 ± 0.33 ng/mL and 1.16 ± 0.59 ng/mL (*P* = 0.0071), respectively. There were significant differences in LH and FSH/LH between the two groups (*P* = 0.0030 and *P* = 0.0005). In contrast, FSH, E2, and DHEA-S levels showed no significant differences between controls and PCOS (*P* > 0.05).

**Table 3:** The blood hormone levels of twenty-two participants in the discovery cohort

	**Controls** **(*n* = 10)**	**PCOS** **(*n* = 12)**	***P*-value**
LH/(IU/L)	7.34 ± 2.05	20.50 ± 5.65	0.0030^*^
FSH/(IU/L)	8.10 ± 5.44	7.76 ± 4.38	0.3016
LH/FSH	0.97 ± 0.39	3.44 ± 1.08	0.0005^*^
E2 (pg/mL)	36.09 ± 16.90	100.24 ± 2.80	0.1277
T/(ng/mL)	0.76 ± 0.33	1.16 ± 0.59	0.0071^*^
DHEA-S (μg/dL)	344.23 ± 170.78	434.81 ± 229.06	0.0851

*LH*, luteinizing hormone; *FSH*, follicle-stimulating hormone; *E2*, estradiol; *T*, testosterone; *DHEA-S*, dehydroepiandrosterone sulfate

*Significant difference between control and PCOS, Student’s *t*-test* P* < 0.05

### Blood RNA editing profiles in controls and PCOS

**Fig. 4:**
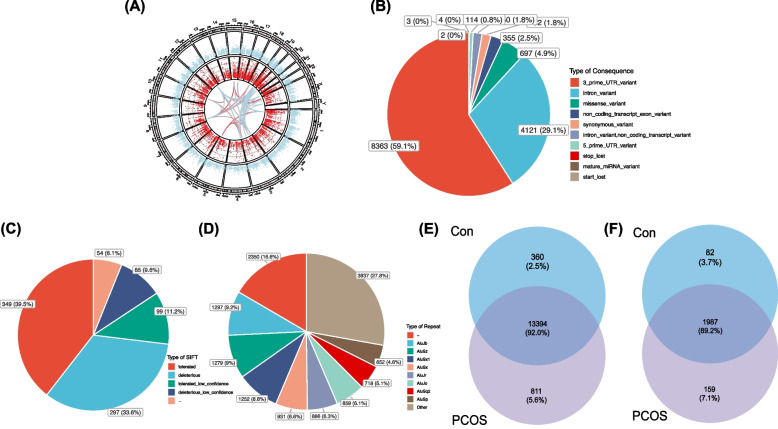
A-to-I RNA editing profiles in peripheral blood samples of PCOS cohort. Twelve PCOS and ten controls are included. **A **A-to-I RNA editing and gene expression across the chromosomes. Red and blue dots represent individual RNA editing events and genes, respectively. **B** The distribution of A-to-I RNA editing events according to their variant functional categories. **C** Sorting Intolerant from Tolerant (SIFT) prediction of missense A-to-I RNA editing variants. **D** Distribution of Alu repetitive elements with A-to-I RNA editing in all peripheral blood samples. **E**–**F** Venn plots comparing A-to-I RNA edited genes and editing events in PCOS and controls

RNA-Seq and further analysis were performed to study the differences in gene expression and RNA editing in blood samples between controls and PCOS. The RNA editing level and editing event density across the human chromosomes observed in all blood samples are shown in Fig. 4A. For the editing events, the main consequence types were 3′-UTR (58.2%), intron (28.5%), and missense variants (6.1%) (Fig. 4B). And 382 missense editing events were predicted to be deleterious (or low confidence deleterious) by SIFT (Fig. 4C). Notably, most of these editing events were located in Alu repetitive elements (Fig. 4D). With 14,565 editing events in 2228 edited genes in all samples, the Venn plots compared the editing events and edited genes between controls and PCOS (Fig. 4E, F).

### Identification of hub RNA editing modules in peripheral blood of PCOS and controls

**Fig. 5:**
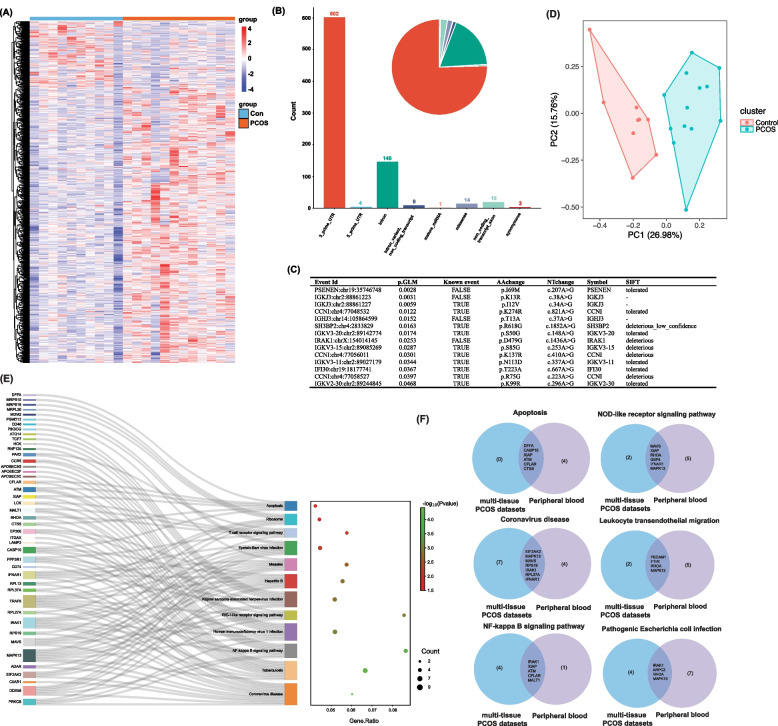
Differential A-to-I RNA editing in peripheral blood samples of PCOS cohort. **A** Heatmap of 798 differential editing events comparing the A-to-I RNA editing patterns between PCOS and controls. **B** The distribution of differential A-to-I RNA editing events according to their variant functional categories, according to annotation by the Variant Effect Predictor. **C** The detailed information of 14 missense differential A-to-I RNA editing events. Known sites are annoted in the REDIportal database. AA change and NTchange are changes in amino acid and nucleotide sequences. **D** PCA based on differential A-to-I RNA editing events in blood samples. **E** KEGG pathway enrichment results on 334 differentially A-to-I RNA edited genes. **F** The overlapped pathways of differentially A-to-I RNA edited genes between peripheral blood samples and public datasets. PCA, principal component analysis; KEGG, Kyoto Encyclopedia of Genes and Genomes

Then, based on the criterion of GLM *P* < 0.05, 798 differential editing events were identified (Fig. 5A, Additional file 2: Table S9). The most abundant of these variants were 3′-UTR variants (75.4%), followed by intronic variants (18.3%) (Fig. 5B). In addition, 14 missense editing events were found (Fig. 5C). PCA using all these DRE events showed that samples from the two groups could be separated clearly (Fig. 5D).

Functional analysis was performed on a total of 334 DEGs to gain insights into the biological processes and pathways altered in PCOS related to RNA editing dysregulation. KEGG analysis revealed several significantly enriched pathways related to viral infections, immune response, and cellular processes (Fig. 5E). GO analysis suggests that neutrophil degranulation and innate immune response were among the most enriched biological processes. Cellular components, such as the secretory granule membrane, lysosomal lumen, and vacuolar lumen, were also enriched by these DEGs. Molecular functions such as RNA binding, cytokine receptor activity, and protein serine/threonine kinase activity were significantly enriched (Additional file 5: Fig S4). Moreover, we found some common DRE-enriched pathways in our blood samples and multi-tissue PCOS cohorts in the public datasets, including coronavirus disease, NF-kappa B signaling pathway, NOD-like receptor signaling pathway, leukocyte transendothelial migration, pathogenic *Escherichia coli* infection, and apoptosis, which underscored their potential significance in the pathogenesis of PCOS (Fig. 5F). Our findings suggest that the dysregulation of RNA editing events in these pathways may contribute to the development and progression of PCOS and warrant further investigation.

**Fig. 6:**
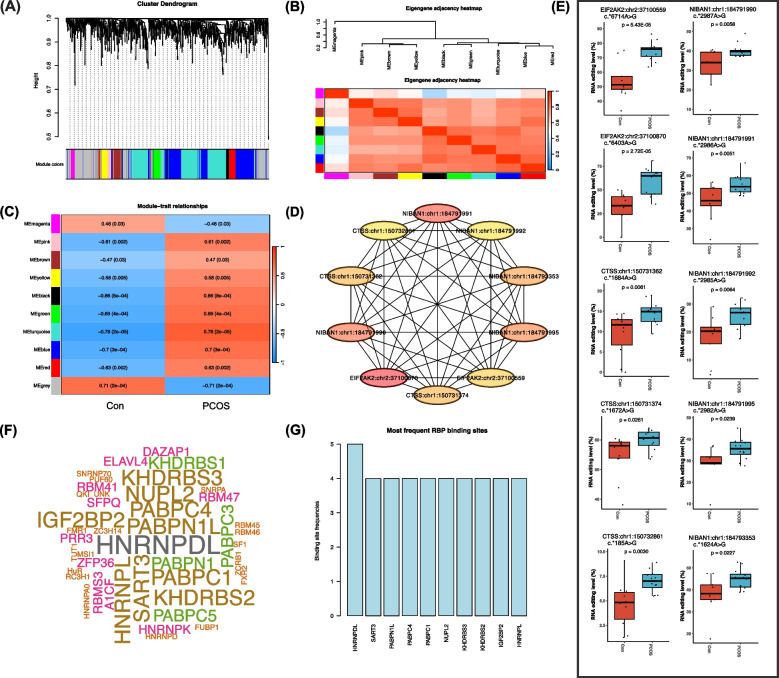
Weighted gene co-expression network analysis (WGCNA) of differential A-to-I RNA editing events in blood samples of PCOS cohort. **A** Sample clustering dendrogram of PCOS and controls. **B** Plot of the eigengene adjacency. **C** Module–trait relationships in PCOS and controls. The color scale denotes the corresponding correlation coefficient and *P*-value. **D** The top ten or hub A-to-I RNA editing events identified through the degree centrality algorithm in Cytoscape within the turquoise module. **E** Bar plots showing the A-to-I RNA editing levels of these ten hub events. **F**, **G** Predictions of RNA-binding proteins (RBPs) that potentially interact with these ten hub A-to-I RNA editing events

WGCNA was further conducted to identify the hub differential editing events in blood samples. The cluster dendrogram in Fig. 6A shows the clustering of the differential editing events in blood samples. The eigengene adjacency heatmap of co-editing modules revealed relationships among these editing events (Fig. 6B). Among the modules obtained, the turquoise module was identified as the key module exhibiting the most significant association with PCOS (*R* = 0.78, *P* = 3.0 × 10^−4^) (Fig. 6C). The module contained 238 nodes and 3335 edges. The degree centrality algorithm implemented in Cytoscape identified the top ten nodes (DRE events) as the hub differential editing events (Fig. 6D, Table 4). The editing levels of these ten DRE events in the blood samples are shown in Fig. 6E.

−

**Table 4:** The top ten hub blood differential RNA editing events associated with PCOS

Event ID	p.GLM	Known event^a^	Consequence^b^	Gene biotype	Gene symbol
EIF2AK2:chr2:37,100,559	5.43E-05	TRUE	3_prime_UTR	protein_coding	*EIF2AK2*
EIF2AK2:chr2:37,100,870	0.0003	TRUE	3_prime_UTR	protein_coding	*EIF2AK2*
CTSS:chr1:150,732,861	0.003	TRUE	3_prime_UTR	protein_coding	*CTSS*
NIBAN1:chr1:184,791,991	0.0051	TRUE	3_prime_UTR	protein_coding	*NIBAN1*
NIBAN1:chr1:184,791,990	0.0058	TRUE	3_prime_UTR	protein_coding	*NIBAN1*
CTSS:chr1:150,731,362	0.0061	TRUE	3_prime_UTR	protein_coding	*CTSS*
NIBAN1:chr1:184,791,992	0.0064	TRUE	3_prime_UTR	protein_coding	*NIBAN1*
NIBAN1:chr1:184,793,353	0.0227	TRUE	3_prime_UTR	protein_coding	*NIBAN1*
NIBAN1:chr1:184,791,995	0.0239	TRUE	3_prime_UTR	protein_coding	*NIBAN1*
CTSS:chr1:150,731,374	0.0261	TRUE	3_prime_UTR	protein_coding	*CTSS*

^a^Recorded in the REDI portal database

^b^Consequence, the variant functional category of editing events in peripheral blood samples

All these ten hub DRE events were identified to be located in the 3′-UTR (Table 4). Forty-four RBPs were predicted to bind to these ten events, and the RBP Heterogeneous Nuclear Ribonucleoprotein D Like (HNRNPDL) was predicted to bind with five of these hub blood DRE events (Fig. 6F, G).

### Correlation between hub DRE events with clinical features and the identification of event *EIF2AK2*:chr2:37,100,559

**Fig. 7:**
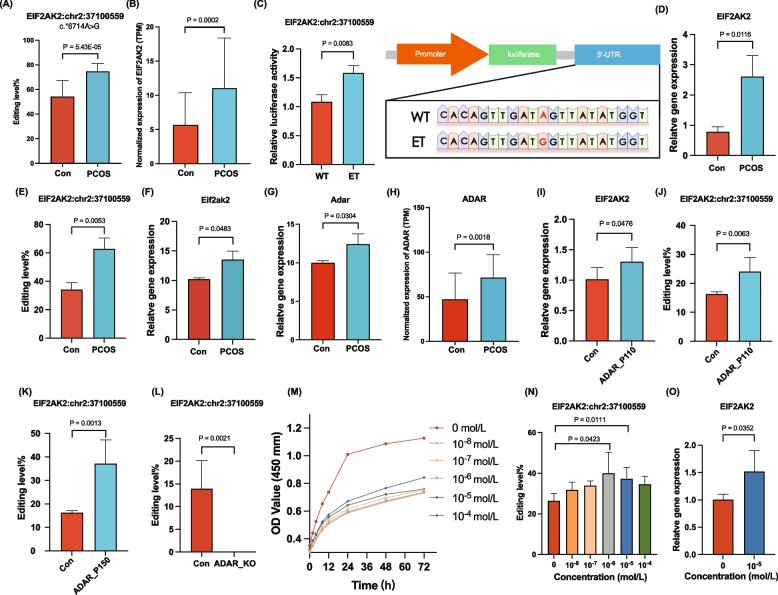
Functional validation for a hub DRE event of *EIF2AK2*:chr2:37,100,559 mediated by ADAR. **A** Bar plot showing the A-to-I RNA editing level of *EIF2AK2*:chr2:37,100,559 in PCOS and controls. **B** Bar plot showing the normalized expression level of *EIF2AK2* in PCOS and controls. **C** Dual-luciferase reporter assays of wild-type compared to the edited-type 3′-UTR surrounding *EIF2AK2*:chr2:37,100,559. **D** Expression level of *EIF2AK2* in the validation cohort quantified by qPCR (*n* = 3). **E** The A-to-I RNA editing level of site *EIF2AK2*:chr2:37,100,559 in the validation cohort quantified by Sanger sequencing (*n* = 3). **F,G ***Eif2ak2* and *Adar* expression in ovarian samples from a prenatally androgenized mouse model. **H ***ADAR* expression in the blood samples of PCOS cohort. **I**–**K ***EIF2AK2* expression and *EIF2AK2*:chr2:37,100,559 editing in HEK293T cells overexpressing ADAR-p110 and ADAR-p150. **L** The A-to-I RNA editing level at *EIF2AK2*:chr2:37,100,559 upon *ADAR* knockout (ADAD_KO). Expression data are obtained from RNA-Seq of A549 cells (PRJNA386593). **M** Proliferation of KGN cells stimulated with DHEA at different concentrations measured using the CCK‐8 method. **N ***EIF2AK2*:chr2:37,100,559 editing after 72 h of DHEA stimulation at varying concentrations. **O** *EIF2AK2* expression after 72 h of DHEA stimulation at 10^−5^ mol/L. TPM, transcript per million; KEGG, Kyoto Encyclopedia of Genes and Genomes

Six clinical hormone parameters of PCOS, namely FSH, LH, LH/FSH, testosterone, DHEA-S, and estradiol, were included in the calculation of the correlation with hub DRE events. *EIF2AK2*:chr2:37,100,559, *EIF2AK2*:chr2:37,100,870, and *NIBAN1*:chr1:184,791,992 were correlated with the LH and LH/FSH levels (Additional file 6: Fig S5). As for these three events, we found site *EIF2AK2*:chr2:37,100,559 showed a positive correlation between the levels of RNA editing and gene expression (Fig. 7A, B, and Additional file 7: Fig S6A). Besides, *EIF2AK2*:chr2:37,100,559 was also one of the top ten events in random forest analysis (Additional file 7: Fig S6B, C). The variant was not detected in the genomic DNA (Additional file 8: Fig S7). In addition, Table 5 showed the hyper-editing of gene *EIF2AK2* in public PCOS RNA-Seq datasets (Table 5), indicating that *EIF2AK2* editing could play a role in various tissues. This suggests that despite the variability in specific editing events, the differential RNA editing of *EIF2AK2* may have significant functional implications in PCOS.

**Table 5:** Hyper-editing of the differential *EIF2AK2* RNA editing events in the blood samples of our PCOS cohort and public PCOS datasets

**Blood samples**	**Endometrium** (**PRJNA938949)**	**Placenta (PRJNA645705)**	**Granulosa cells (PRJNA794860)**
*EIF2AK2*:chr2:37100426	*EIF2AK2*:chr2:37100471	*EIF2AK2*:chr2:37100527	*EIF2AK2*:chr2:37100446
*EIF2AK2*:chr2:37100471	*EIF2AK2*:chr2:37100873	*EIF2AK2*:chr2:37100954	*EIF2AK2*:chr2:37100523
*EIF2AK2*:chr2:37100559	*EIF2AK2*:chr2:37100944	*EIF2AK2*:chr2:37102071	*EIF2AK2*:chr2:37100527
*EIF2AK2*:chr2:37100857	*EIF2AK2*:chr2:37102068	*EIF2AK2*:chr2:37102140	*EIF2AK2*:chr2:37103471
*EIF2AK2*:chr2:37100858	*EIF2AK2*:chr2:37102226		*EIF2AK2*:chr2:37104058
*EIF2AK2*:chr2:37100869	*EIF2AK2*:chr2:37103248		*EIF2AK2*:chr2:37104134
*EIF2AK2*:chr2:37100870	*EIF2AK2*:chr2:37103258		
*EIF2AK2*:chr2:37100873	*EIF2AK2*:chr2:37103288		
*EIF2AK2*:chr2:37100954	*EIF2AK2*:chr2:37103360		
*EIF2AK2*:chr2:37101540	*EIF2AK2*:chr2:37103477		
*EIF2AK2*:chr2:37101955	*EIF2AK2*:chr2:37103946		
*EIF2AK2*:chr2:37102157	*EIF2AK2*:chr2:37104064		
*EIF2AK2*:chr2:37103468	*EIF2AK2*:chr2:37104138		
*EIF2AK2*:chr2:37103934	*EIF2AK2*:chr2:37104404		
*EIF2AK2*:chr2:37104499	*EIF2AK2*:chr2:37104497		
*EIF2AK2*:chr2:37104786			
**Granulosa cells (PRJNA707301)**	**T helper cells (PRJNA540679)**	**Abdominal adipose tissues (PRJNA798018Ab)**	**Gluteofemoral adipose tissues (PRJNA798018GF)**
*EIF2AK2*:chr2:37102140	*EIF2AK2:*chr2:37102118	*EIF2AK2*:chr2:37100954	*EIF2AK2*:chr2:37102118
		*EIF2AK2*:chr2:37100944	*EIF2AK2*:chr2:37104497
		*EIF2AK2*:chr2:37100873	

Furthermore, the validation cohort also demonstrated a heightened editing level of the *EIF2AK2*:chr2:37,100,559 and expression level of *EIF2AK2* in individuals with PCOS (Fig. 7D, E). In mouse models, the gene *Eif2ak2* also expressed a significantly higher level in ovarian samples from prenatal androgenized mice (Dataset PRJNA659669) (Fig. 7F).

### Altered *EIF2AK2* expression was regulated by ADAR*-*mediated cis-RNA editing

To further validate the correlation between RNA editing at the *EIF2AK2*:chr2:37,100,559 site and *EIF2AK2* gene expression, we performed dual luciferase reporter assays in HEK 293 T cells. A significant increase of luciferase activity was found in cells transfected with a plasmid containing the edited *EIF2AK2* site than in cells with the wild-type plasmid (Fig. 7C), indicating that RNA editing at *EIF2AK2*:chr2:37,100,559 could increase *EIF2AK2* expression.

We also observed that the expression of *ADAR* was significantly elevated in PCOS (Fig. 7G). Overexpression of ADAR-p110 and ADAR-p150 plasmids in HEK 293 T cells upregulated expression of *EIF2AK2* and the RNA editing level of *EIF2AK2*:chr2:37,100,559 (Fig. 7H), which were confirmed by Sanger sequencing (Fig. 7I, J). Moreover, the analysis of public RNA-Seq data (PRJNA386593) showed a significant decrease in *EIF2AK2* expression and *EIF2AK2*:chr2:37,100,559 editing upon *ADAR* knockout (Fig. 7K), suggesting the editing site *EIF2AK2*:chr2:37,100,559 was directly induced by *ADAR*.

After 72 h of stimulation with DHEA at varying concentrations (0, 10^−8^, 10^−7^, 10^−6^,10^−5^, 10^−4^ mol/L), we found that the editing level of site *EIF2AK2* at chr2:37,100,559 and expression level of *EIF2AK2* were significantly upregulated in the 10^−5^ mol/L group (Fig. 7L, M). Meanwhile, the DHEA treatment also decreased the cell proliferative capability (Fig. 7N).

Previous studies have revealed that PCOS patients are often in an inflammatory state with a higher level of LPS [63, 64]. A previous study has revealed that LPS stimulation could induce KGN cell inflammation [65]. To further explore the relationship between LPS and *EIF2AK2*, we stimulated KGN cells with 1 μg/mL LPS for 24 h and found that *EIF2AK2* and inflammatory cytokines (IL-6, IL-18, and TNF-α) were upregulated (Additional file 9: Fig S8A–D). This finding was consistent with public dataset (PRJNA993124) results showing increased *EIF2AK2* expression in THP-1 cells after LPS stimulation (Additional file 9: Fig S8E).

### Role of differential editing in PCOS diagnosis

We also utilized ROC curves to assess the diagnostic accuracy of differential RNA editing. Dataset PRJNA719824, with the largest sample size (Con: 96, PCOS: 84), was used for ROC analysis. We identified five differential RNA editing events, *ERCC2*:chr19:45,350,155, *ALDH6A1*:chr14:74,058,840, *LONP2*:chr16:48,354,269, *MDM2*:chr12:68,843,263, and *AC008764.4*:chr19:16,634,547, which were present in at least three datasets including PRJNA719824 (with the largest volume of samples) and were used to construct ROC curves. The AUCs for these five RNA editing events were 0.6468, 0.6606, 0.6013, 0.6436, and 0.6781, respectively (Additional file 10: Fig S9A–E). Combining these five events resulted in a higher AUC of 0.7655 (Additional file 10: Fig S9F). *EIF2AK2*:chr2:37,100,559, with an AUC of 0.9167 in our peripheral blood samples, emerged as a particularly promising diagnostic marker, as presented in Additional file 10: Fig S9G.

### Potential function of gene *EIF2AK2* in PCOS

**Fig. 8:**
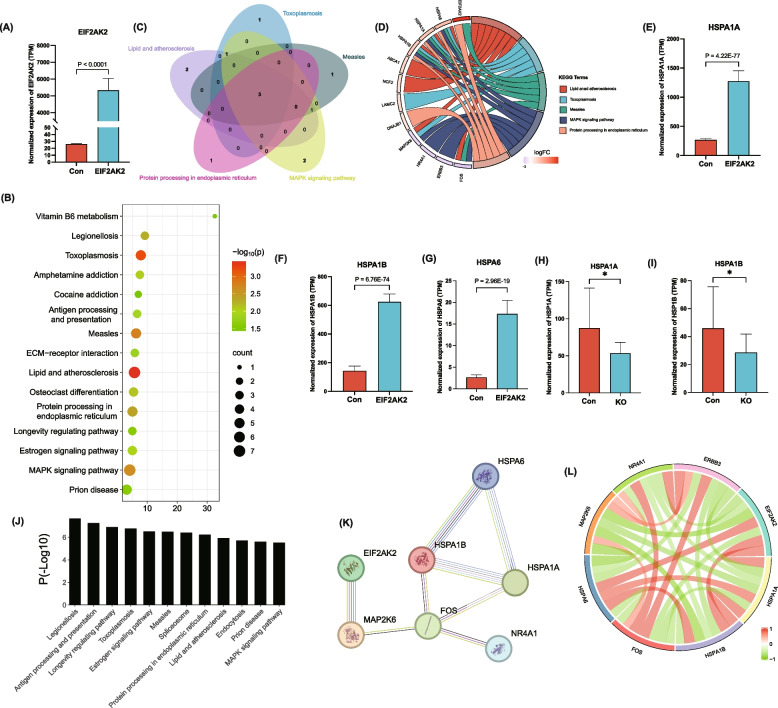
Functional exploration of the role played by *EIF2AK2* in PCOS. **A ***EIF2AK2* overexpression in HeLa cells (PRJNA554006). **B** KEGG enrichment of 123 differentially expressed genes based on the criteria of FDR ≤ 0.05, |logFC|≥ 1 in HeLa cells upon *EIF2AK2* overexpression (PRJNA554006). **C**, **D** Common genes in the top five pathways. Three genes, including *HSPA1A*, *HSPA1B*, and *HSPA6* are shared by these five pathways. **E**–**G** Expression levels of *HSPA1A*, *HSPA1B*, and *HSPA6* in HeLa cells upon *EIF2AK2* overexpression (PRJNA554006). **H**, **I** Expression of *HSPA1A* and *HSPA1B* in A549 cells upon *EIF2AK2* knockout (KO) (PRJNA850448). **J** Functional analysis of three common genes in the top five pathways. **K** PPI network showing the interaction between *EIF2AK2* and MAPK pathway-related genes. **L** Expression correlations between *EIF2AK2* and MAPK pathway-related genes in HeLa cells resulting from the *Pearson* method. logFC, log of fold change; FDR, false discovery rate; PPI, protein–protein interactive

To determine the functional role of *EIF2AK2* in PCOS, we undertook a comprehensive analysis of the publicly available dataset PRJNA554006 which consists of two *EIF2AK2* overexpression and two control HeLa cells samples. Figure 8A shows the expression of *EIF2AK2* after transfection. Based on the threshold of FDR ≤ 0.05 and |logFC| ≥ 1, a total of 123 differentially expressed genes (55 downregulated and 68 upregulated) were identified in response to *EIF2AK2* overexpression (Additional file 11: Fig S10A, Additional file 2: Table S10). Lipid and atherosclerosis, toxoplasmosis, measles, protein processing in the endoplasmic reticulum pathway, and the MAPK signaling pathway were the most significantly enriched pathways (Fig. 8B–D). Three heat shock protein family A genes, including 1A (*HSPA1A*), 1B (*HSPA1B*), and 6 (*HSPA6*) were overlapped in these five pathways, and confirmed to be significantly down-regulated in A549 lung epithelial cells with *EIF2AK2* knockout (PRJNA850448), among which *HSPA1A* and *HSPA1B* showed significant and opposite changes observed in HeLa cells (Fig. 8E–I). KEGG results showed that these genes were mainly involved in antigen processing and presentation, estrogen signaling pathway, and MAPK signaling pathway (Fig. 8J). The MAPK pathway has been reported to play a pivital role in PCOS [66]. GSEA analysis of these 123 differentially expressed genes also showed the MAPK pathway was one of the key upregulated pathways in response to *EIF2AK2* overexpression (Additional file 11: Fig S10B, C). Protein–protein interactive (PPI) network analysis also showed the interaction between *EIF2AK2* and MAPK pathway-related genes (Fig. 8L-K). Our study suggests the potential linkage between *EIF2AK2* and the MAPK signaling pathway, and further experimental validation is warranted.

## Discussion

RNA editing, a post-transcriptional modification, has been shown to play an important role in various biological processes and disease developments [67, 68]. Our previous study identified the link between A-to-I RNA editing in granulosa cells and PCOS [32]. In our current study, we investigated A-to-I RNA editing in PCOS based on public datasets of multiple tissues and our peripheral blood samples at the transcriptome level to provide insight into the potential involvement of RNA editing in PCOS pathogenesis.

Our analysis of the public RNA-Seq datasets underlined substantial and tissue-specific RNA editing changes in PCOS and identified six consistent DRE events in at least four of the twelve datasets. By far, there has been only limited research on these editing events, highlighting the need for further investigation to understand their potential role in the pathogenesis of PCOS. Delta 4-desaturase and sphingolipid 1 (*DEGS1*) are involved in the sphingolipid metabolism and adipocyte differentiation [69, 70]. Tubulin alpha 1b (*TUBA1B*), encoding the alpha-tubulin protein, is involved in microtubule formation and has been reported to play a role in several biological processes, including mismatch repair and cell cycle pathway [71, 72]. Previous studies have also suggested that abnormal *TUBA1B* expression and RNA editing could be linked to meiosis or PCOS [32, 73]. As for tripartite motif containing 56 (*TRIM56*), previous studies have shown that it plays a crucial role in regulating immune response and viral defense by promoting the activation of innate immunity and inducing expression of interferon-stimulated genes [74, 75]. Moreover, serine/threonine kinase 4 (*STK4*) has been reported to be involved in regulating apoptosis and cell proliferation [76, 77]. Excision repair cross-complementation group 2 (*ERCC2*) is engaged in nucleotide excision repair, a crucial DNA repair pathway that protects DNA against the harmful effects of UV radiation and other DNA-damaging agents [78, 79]. *ERCC2* SNPs have been associated with male infertility [80]. Zinc finger protein 83 (*ZNF83*), a member of the zinc finger protein family, has been linked to cancer through its mutation that has been shown to promotes the activation of NF-κB [81]. However, the exact functional relavance of these editing events in PCOS remains to be elucidated.

The consistent DRE events identified in granulosa cells across multiple datasets might offer significant insights into the biological mechanisms related to ovarian function in PCOS. Granulosa cells are a crucial component of the ovarian follicle and plays a vital role in regulating steroid hormone synthesis [82, 83]. Apoptosis is a key process involved in granulosa cell function, and has been shown to play essential roles in the pathogenesis of PCOS [84, 85]. Our previous study found that the function enrichment of differentially edited genes in granulosa cells was also related to apoptosis [32]. Our observation suggests that RNA editing may be involved in the regulation of apoptosis-related processes in granulosa cells in PCOS.

Moreover, functional enrichement analysis also demonstrates the potential significance of differentially edited genes in multiple tissues of PCOS. Common pathways were enriched in both our peripheral blood samples and other tissue types in public datasets. The results suggest that RNA editing, which is an epigenetic mechanism, is possibly involved in the dysregulation of immune and inflammatory responses in PCOS, as suggested by the enriched pathways such as NF-kappa B signaling and NOD-like receptor signaling. This is consistent with previous studies that have reported associations between PCOS and chronic low-grade systemic inflammation and immune dysfunction [86–89]. Again, the enrichment of the apoptosis pathway indicates that the balance between cell survival and cell death may be disturbed in PCOS, which could lead to abnormal follicular development and other related symptoms, emphasizing that apoptosis plays a crucial role in the pathogenesis of PCOS. Dysregulation of the PI3K/Akt signaling pathway, oxidative stress, and inflammation can lead to granulosa cell apoptosis in PCOS, which may contribute to the development of ovarian dysfunction and endometrial hyperplasia, a precursor to endometrial cancer [85]. Glycolytic enzymes and mitochondrial-dysfunction could also contribute to apoptosis in PCOS patients with endometrial hyperplasia [90]. Additionally, microRNA-21 has been shown to regulate apoptosis and cell proliferation in PCOS granulosa cells, further supporting the role of apoptosis in PCOS pathogenesis [91]. Apoptosis could be a potential therapeutic target for PCOS treatment. These findings provide new insights into the potential molecular mechanisms underlying the pathogenesis of PCOS and suggest potential therapeutic targets for it.

Our results also indicate that a role of RBPs in the pathophysiology of PCOS as they were predicted to bind to the DRE sites [92]. HNRNPDL plays a critical role in regulating transcription and alternative splicing [93]. It has also been implicated in several muscular dystrophies, including HNRNPDL-related muscular dystrophy with varying clinical and MRI phenotypes [94, 95]. Additionally, HNRNPDL is found to be the most coexpressed RBP with alternative splicing events in severe acute pancreatitis [96]. Our findings further suggest the potential involvement of RBPs in the regulation of RNA editing in PCOS. Further studies are needed to elucidate the exact mechanisms by which these RBPs especially HNRNPDL contribute to the development and progression of PCOS.

Dysregulation of *ADAR* expression has been previously implicated in various biological processes and diseases, highlighting its crucial role in RNA editing and gene regulation [97]. However, the role of *ADAR* in PCOS remains unclear. One of the most important targets of ADAR-mediated A-to-I RNA editing identified in our current PCOS study was *EIF2AK2*, which was commonly differentially edited across different PCOS tissues in the public datasets and our peripheral blood samples. *EIF2AK2* encodes protein kinase R (*PKR*), a critical component of the innate immune response [33]. It is involved in defense against viral infections and regulation of cellular stress responses [98, 99] by coordinating the cellular response to viral infections and maintaining homeostasis by controlling mRNA translation [100]. *EIF2AK2* has been considered as a regulator in the systemic lupus erythematosus [33]. Our study further provides evidence supporting a potential link between ADAR-mediated RNA editing and the regulation of *EIF2AK2* activity in PCOS.

As for the function of *EIF2AK2*, the MAPK pathway was identified as one of its downstream targets in PCOS. The MAPK pathway was reported to regulate the granulosa cell apoptosis and the production of steroid biosynthesis in PCOS [101–103]. Therefore, the *EIF2AK2*-MAPK axis could be an important link between epigenetic variation and PCOS phenotype. Further research is needed to thoroughly investigate how *EIF2AK2* regulates the MAPK pathway to better understand the pathogenesis of PCOS and provide potential targets for PCOS diagnosis and treatment.

The field of PCOS epigenetic research is advancing rapidly. To further advance our understanding and treatment of this complex disorder, future epigenetic, especially RNA editing studies, future strategies may focus on (a) Multi-omics data integration. Combining omics data in analysis can reveal complex biological networks and identify new therapeutic targets, deepening our understanding of the multifactorial etiology of PCOS. (b) Prospective and longitudinal studies. Tracking epigenetic changes over time can help determine how they are involved in PCOS development and progression and provide new insight into developing new intervention strategies. (c) Application of RNA editing biomarkers in PCOS precision medicine. Our results also indicated that the use of RNA editing biomarkers could be of importance in developing new strategies of diagnosis and therapies in precision medicine for PCOS.Our findings thus warranted further studies on RNA editing in PCOS.

## Conclusions

In summary, our study provides new insights into the role of RNA editing in the pathogenesis of PCOS, highlighting the RNA editing in *EIF2AK2* and its underlying regulatory mechanisms in the inflammatory feature of the disease. These findings could contribute to our understanding of PCOS etiology and the development of new therapeutic strategies for the disease.

### Supplementary Information

Additional file 1: **Table S1.** The number of differential RNA editing events consequences in different studies. **Table S2.** RNA editing events in different studies.

Additional file 2: **Fig S1.** The principal components of differential editing events reveal the difference in RNA editing patterns between the PCOS and Controls. **Table S3.** Consistent differential editing events in multiple datasets. **Table S4.** Consistent differentially edited genes in multiple datasets. **Table S5.** KEGG functional analysis of consistent differentially edited genes in multiple datasets. **Table S6.** GO Biological Process analysis of consistent differentially edited genes in multiple datasets. **Table S7.** GO Cellular Component analysis of consistent differentially edited genes in multiple datasets. **Table S8.** GO Molecular Function analysis of consistent differentially edited genes in multiple datasets. **Table S9.** Differential editing events between PCOS and Controls in peripheral blood samples. **Table S10.** 123 differentially expressed genes in response to *EIF2AK2* overexpression.

Additional file 3: **Fig S2.** The UpSet plots showing differential editing events (A) and differentially edited genes (B) shared by at least three datasets.

Additional file 4: **Fig S3.** Prediction of RNA-binding proteins (RBPs) that potentially interact with differential editing events identified in at least three separate datasets.

Additional file 5: **Fig S4.** GO analysis of differentially edited genes in peripheral blood between PCOS and controls.

Additional file 6: **Fig S5.** Correlation analysis between the ten hub RNA editing events and clinical hormone indices in PCOS patients.

Additional file 7: **Fig S6.** (A) Analysis of cis-regulatory effects of the editing level of *EIF2AK2*:chr2:37,100,559 on *EIF2AK2* expression. (B) Random Forest analysis identifying the ten hub RNA editing events based on their impact on PCOS phenotype prediction. (C) Venn diagram showing the overlap of significant editing events identified by Random Forest analysis and WGCNA.

Additional file 8: **Fig S7.** Verification of RNA editing event. 10 bp upstream and 10 bp downstream surrounding the editing site (*EIF2AK2*:chr2:37,100,559) in control (A) and PCOS (B) blood samples. 

Additional file 9: **Fig S8.** (A) Expression level of *EIF2AK2* in THP-1 cells 24 h post-stimulation with 1 µg/mL lipopolysaccharide (LPS). (B-D) Expression levels of inflammatory cytokines in response to LPS stimulation. (E) *EIF2AK2* expression in THP-1 cells from dataset PRJNA993124 after LPS stimulation.

Additional file 10: **Fig S9.** ROC curves giving the diagnostic value of editing events in PCOS. (A-E) The AUC value of RNA editing levels of combined *ERCC2*:chr19:45,350,155, *ALDH6A1*:chr14:74,058,840,*LONP2*:chr16:48,354,269, *MDM2*:chr12:68,843,263 and *AC008764.4*:chr19:16,634,547 in dataset PRJNA719824, respectively. (F) The AUC value of the five combined editing events in dataset PRJNA719824. (G) The AUC value of the classifier generated by RNA editing level of *EIF2AK2*:chr2:37,100,559 in peripheral blood samples of PCOS cohort.

Additional file 11: **Fig S10.** (A) Heatmap of 123 differentially expressed genes based on the criteria of FDR ≤ 0.05, |logFC|≥ 1 in dataset PRJNA554006. (B) Analysis of the MAPK signaling pathway via gene set enrichment analysis (GSEA). (C) Expression heatmap of seven MAPK-related genes in dataset PRJNA554006.

## Data Availability

Datasets PRJNA576231 (http://identifiers.org/Bioproject:PRJNA576231), PRJNA649934 (http://identifiers.org/Bioproject:PRJNA649934), PRJNA679416 (http://identifiers.org/Bioproject:PRJNA679416), PRJNA707301 (http://identifiers.org/Bioproject:PRJNA707301), PRJNA794860 (http://identifiers.org/Bioproject:PRJNA794860), PRJNA540679 (http://identifiers.org/Bioproject:PRJNA540679), PRJNA645705 (http://identifiers.org/Bioproject:PRJNA645705), PRJNA719824 (http://identifiers.org/Bioproject:PRJNA719824), PRJNA938949 (http://identifiers.org/Bioproject:PRJNA938949), PRJNA798018 (http://identifiers.org/Bioproject:PRJNA798018), PRJNA386593 (http://identifiers.org/Bioproject:PRJNA386593), PRJNA659669 (http://identifiers.org/Bioproject:PRJNA659669), PRJNA993124 (http://identifiers.org/Bioproject:PRJNA993124), PRJNA554006 (http://identifiers.org/Bioproject:PRJNA554006), and PRJNA850448 (http://identifiers.org/Bioproject:PRJNA850448) are available in the NCBI Gene Expression Omnibus (GEO, http://www.ncbi.nlm.nih.gov/geo) database and European Nucleotide Archive Databank (http://www.ncbi.nlm.nih.gov/geo). The peripheral blood transcriptome sequencing data analyzed in this study are available in the Genome Sequence Archive at the National Genomics Data Center, Beijing Institute of Genomics, Chinese Academy of Sciences / China National Center for Bioinformation (https://ngdc.cncb.ac.cn/gsa/, BioProject: PRJCA019202).

## Abbreviations

3′-UTR: 3′-Untranslated regions
ADAR: Adenosine deaminase, RNA specific
ALDH6A1: Aldehyde dehydrogenase 6 family member A1
ANAPC16: Anaphase-promoting complex subunit 16
A-to-I RNA editing: Adenosine-to-inosine RNA editing
DAZAP1: DAZ-associated protein 1
DEGS1: Delta 4-desaturase and sphingolipid 1
DHEA-S: Dehydroepiandrosterone sulfate
DRE: Differential RNA editing
EIF2AK2: Eukaryotic translation initiation factor 2-alpha kinase 2
ENA: European Nucleotide Archive
ERCC2: Excision repair cross-complementation group 2
FSH: Follicle-stimulating hormone
GLM: Generalized linear model
GO: Gene Ontology
ICMA: Immunochemiluminometric assay
KEGG: Kyoto Encyclopedia of Genes and Genomes
LH: Luteinizing hormone
LPS: Lipopolysaccharide
LRT: Likelihood ratio test
MAVS: Mitochondrial antiviral-signaling protein
MDM2: MDM2 proto-oncogene
NCBI: National Center for Biotechnology Information
PCOS: Polycystic ovary syndrome
RBM45: RNA-binding motif protein 45
RBMS3: RNA-binding motif single-stranded interacting protein 3
RBP: RNA-binding protein
ROC: Receiver operating characteristic
SMIM14: Small integral membrane protein 14
SRA: Sequence Read Archive
STK4: Serine/threonine kinase 4
T: Testosterone
TRIM56: Tripartite motif containing 56
TUBA1B: Tubulin alpha 1b
YBX1: Y-box binding protein 1
ZNF83: Zinc finger protein 83
