# Supplementary material for: Dysregulated RNA editing of EIF2AK2 in polycystic ovary syndrome: clinical relevance and functional implications
Source: BMC Med. 2024 Jun 10;22:229. doi: 10.1186/s12916-024-03434-8 (PMC11163819; doi:10.1186/s12916-024-03434-8)
Supplement: Supplementary file 1 — Additional file 1: Table S1. The number of differential RNA editing events consequences in different studies. Table S2. RNA editing events in different studies. [file 12916_2024_3434_MOESM1_ESM.docx]

**Table S1.**

The number of differential RNA editing events consequences in different studies.

| **Study ID** | **3 Prime UTR** | **5 Prime UTR** | **Intron** | **Noncoding Transcript** | **Intron_ Noncoding** | **Synonymous** | **Missense** |
| --- | --- | --- | --- | --- | --- | --- | --- |
| PRJNA540679 | 28 | - | 59 | 3 | 3 | - | 2 |
| PRJNA576231 | 362 | 4 | 136 | 25 | 15 | 2 | 2 |
| PRJNA645705 | 385 | 7 | 227 | 25 | 27 | 4 | 5 |
| PRJNA649934_GC | 92 | 53 | 35 | 20 | 5 | 10 | 26 |
| PRJNA649934_OC | 137 | 28 | 135 | 51 | 69 | 12 | 21 |
| PRJNA679416 | 6 | - | - | 2 | 1 | 1 | 1 |
| PRJNA707301 | 544 | 3 | 3980 | 30 | 131 | 2 | - |
| PRJNA719824 | 94 | 2 | 20 | 2 | - | 1 | 3 |
| PRJNA794860 | 869 | 11 | 520 | 30 | 39 | 1 | 7 |
| PRJNA938949 | 2469 | 15 | 12594 | 140 | 836 | 3 | 12 |
| PRJNA798018_ABD | 101 | - | 31 | 4 | 8 | 2 | - |
| PRJNA798018_GF | 88 | - | 36 | 2 | 9 | 2 | - |

Intron_ Noncoding: intron_variant,non_coding_transcript_variant

Noncoding Transcript: non_coding_transcript_exon_variant

**Table S2.**

RNA editing events in different studies.

| **Study ID** | **Editing Events** | **Editing Genes** |
| --- | --- | --- |
| PRJNA540679 | 2517 | 828 |
| PRJNA576231 | 17395 | 3644 |
| PRJNA645705 | 12042 | 4237 |
| PRJNA649934_GC | 3933 | 2236 |
| PRJNA649934_OC | 8917 | 3539 |
| PRJNA679416 | 925 | 616 |
| PRJNA707301 | 205015 | 7566 |
| PRJNA719824 | 6305 | 1163 |
| PRJNA794860 | 14707 | 3601 |
| PRJNA938949 | 137722 | 7519 |
| PRJNA798018_ABD | 2711 | 655 |
| PRJNA798018_GF | 2656 | 645 |
