# Supplementary figures and images for: Dysregulated RNA editing of EIF2AK2 in polycystic ovary syndrome: clinical relevance and functional implications
Source: BMC Med. 2024 Jun 10;22:229. doi: 10.1186/s12916-024-03434-8 (PMC11163819; doi:10.1186/s12916-024-03434-8)

PRJNA540679

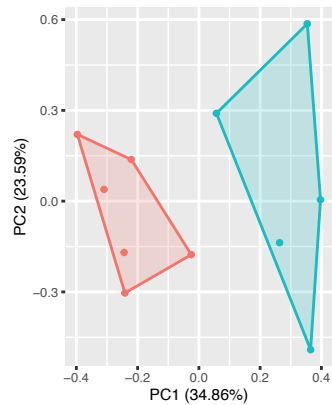

PRJNA576231

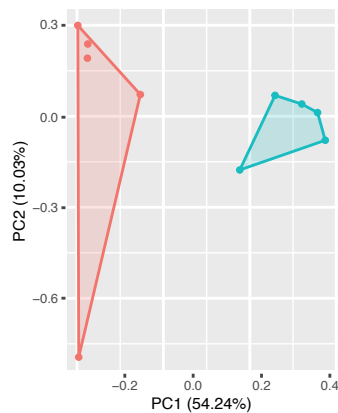

PRJNA645705

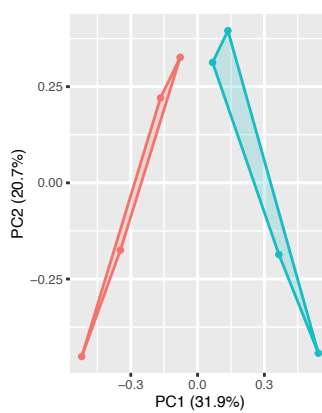

PRJNA649934\_GC

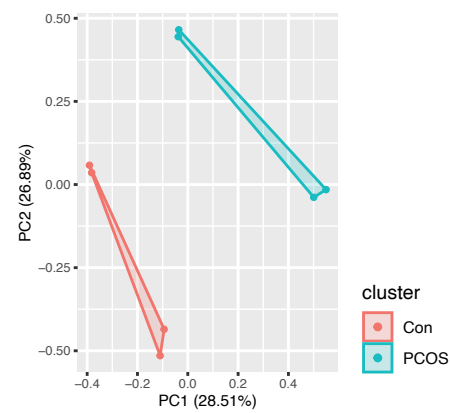

PRJNA649934\_OC

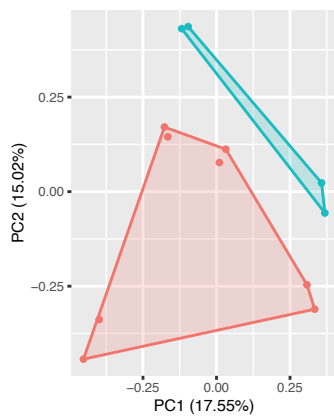

PRJNA679416

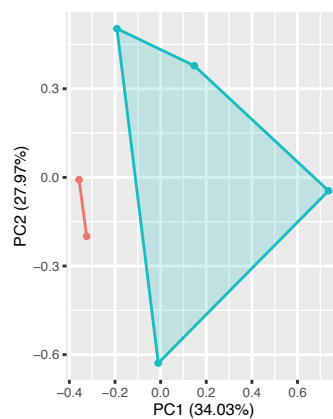

PRJNA707301

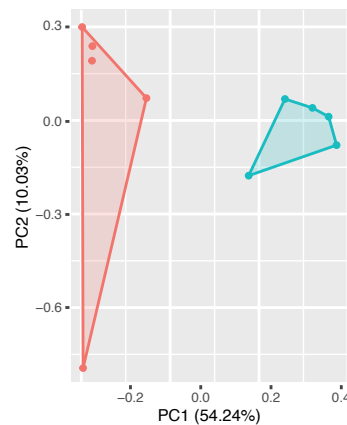

PRJNA719824

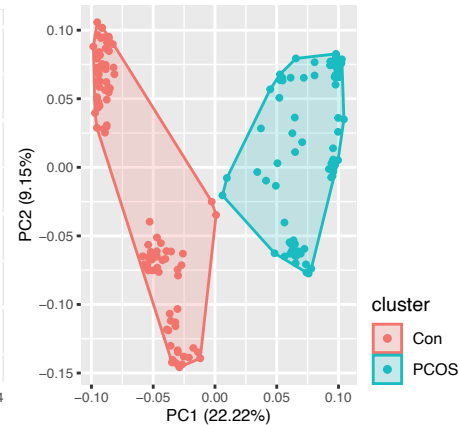

PRJNA794860

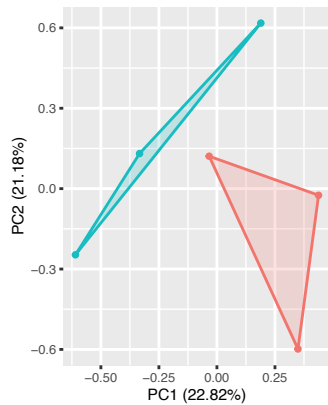

PRJNA798018\_ABD

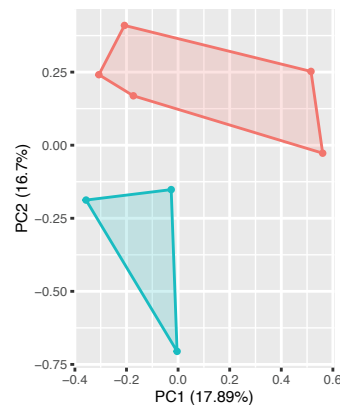

PRJNA798018\_GF

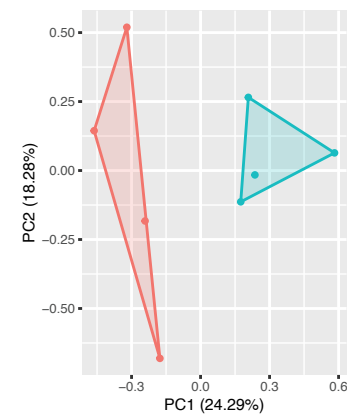

PRJNA938949

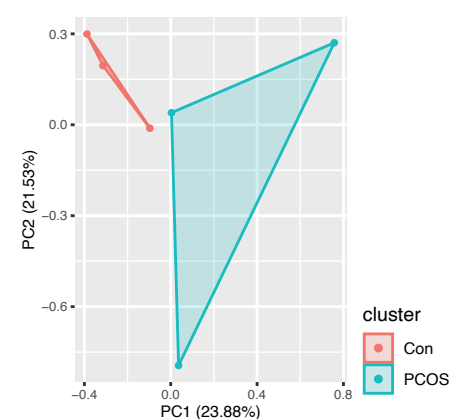

Supplement: Supplementary file 2 — Additional file 2: Fig S1. The principal components of differential editing events reveal the difference in RNA editing patterns between the PCOS and Controls. Table S3. Consistent differential editing events in multiple datasets. Table S4. Consistent differentially edited genes in multiple datasets. Table S5. KEGG functional analysis of consistent differentially edited genes in multiple datasets. Table S6. GO Biological Process analysis of consistent differentially edited genes in multiple datasets. Table S7. GO Cellular Component analysis of consistent differentially edited genes in multiple datasets. Table S8. GO Molecular Function analysis of consistent differentially edited genes in multiple datasets. Table S9. Differential editing events between PCOS and Controls in peripheral blood samples. Table S10. 123 differentially expressed genes in response to EIF2AK2 overexpression. [file 12916_2024_3434_MOESM2_ESM.zip › Supplementary Figure 1_ESM.pdf]

(A)

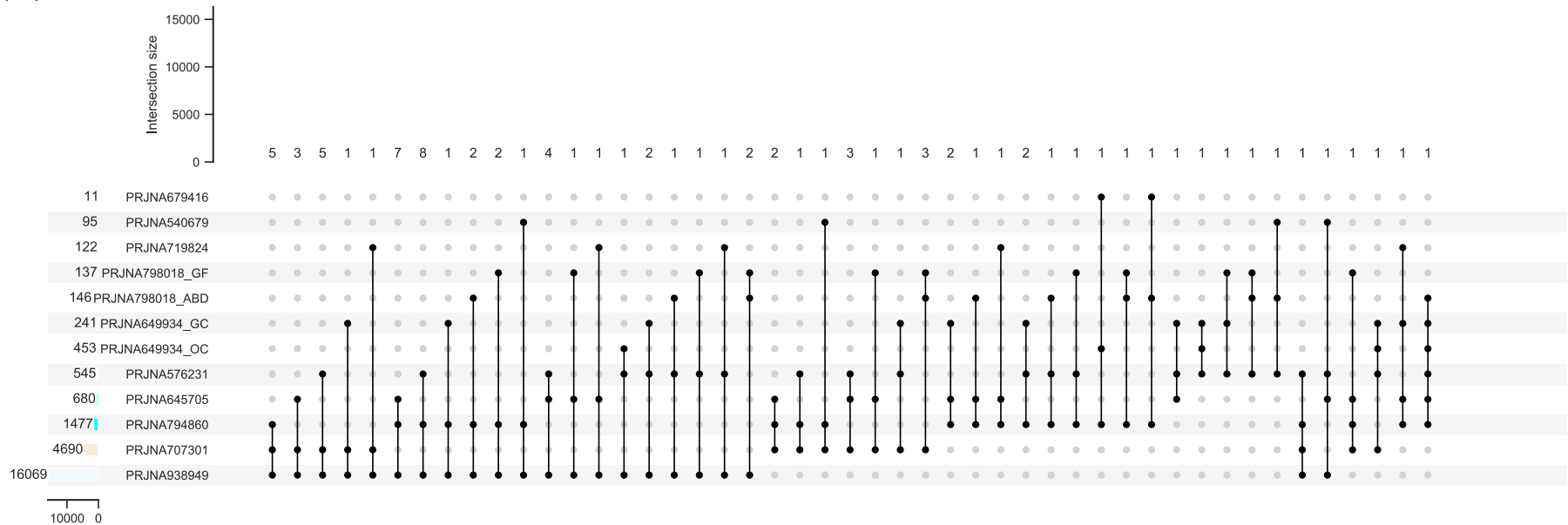

**(B)**

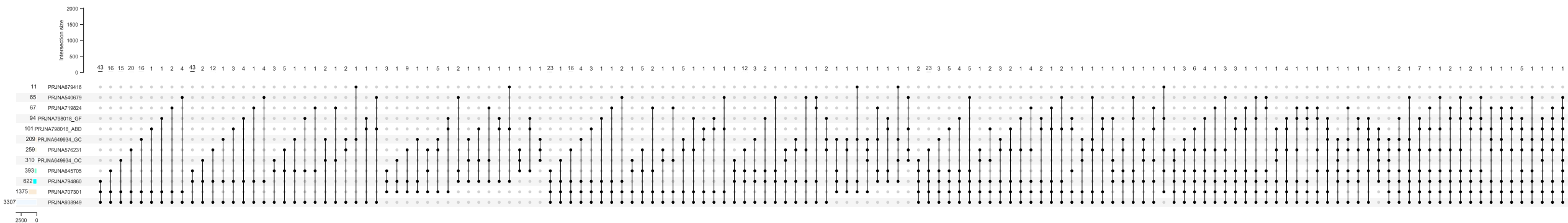

Supplement: Supplementary file 3 — Additional file 3: Fig S2. The UpSet plots showing differential editing events (A) and differentially edited genes (B) shared by at least three datasets. [file 12916_2024_3434_MOESM3_ESM.pdf]

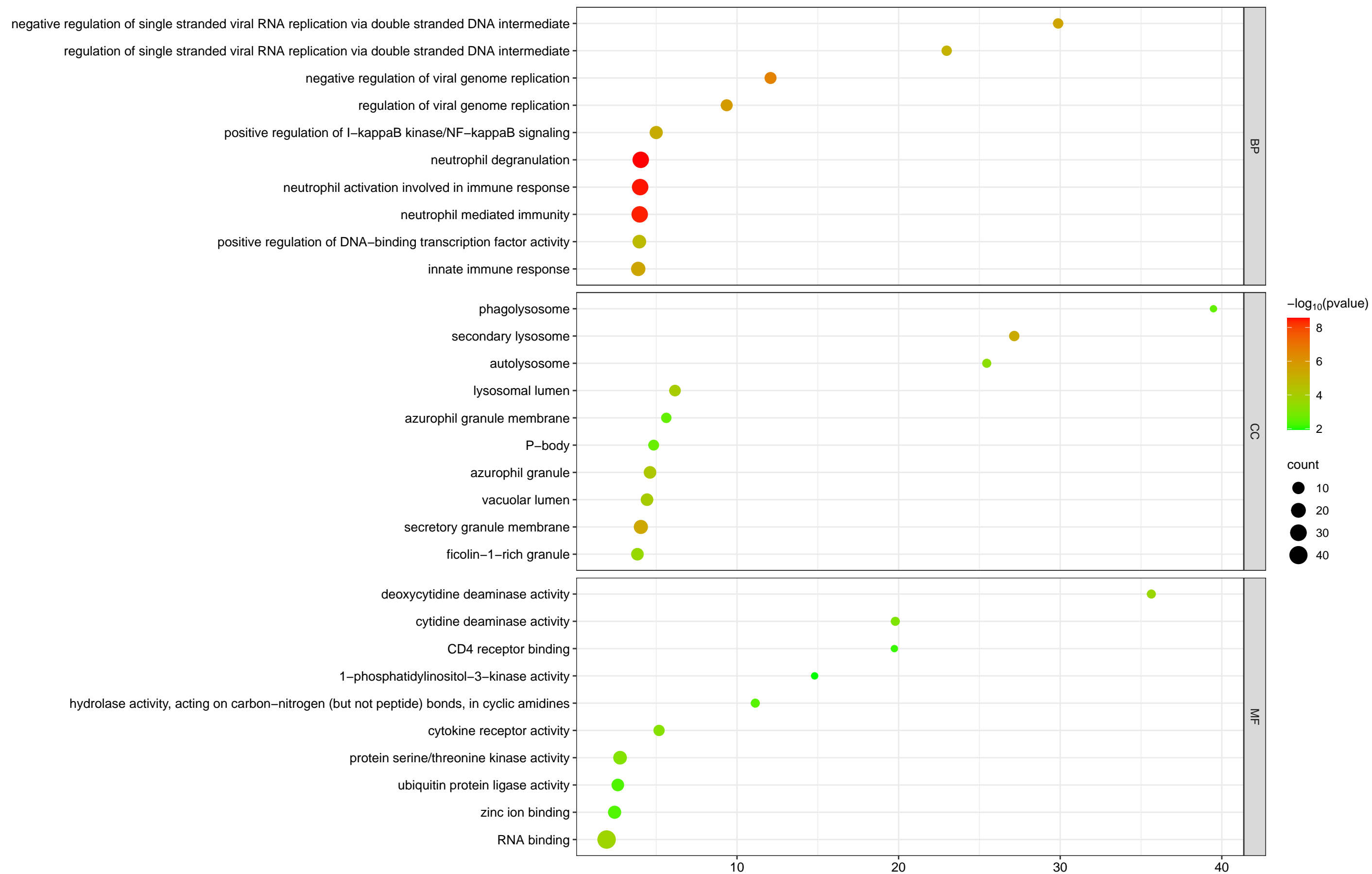

Supplement: Supplementary file 5 — Additional file 5: Fig S4. GO analysis of differentially edited genes in peripheral blood between PCOS and controls. [file 12916_2024_3434_MOESM5_ESM.pdf]

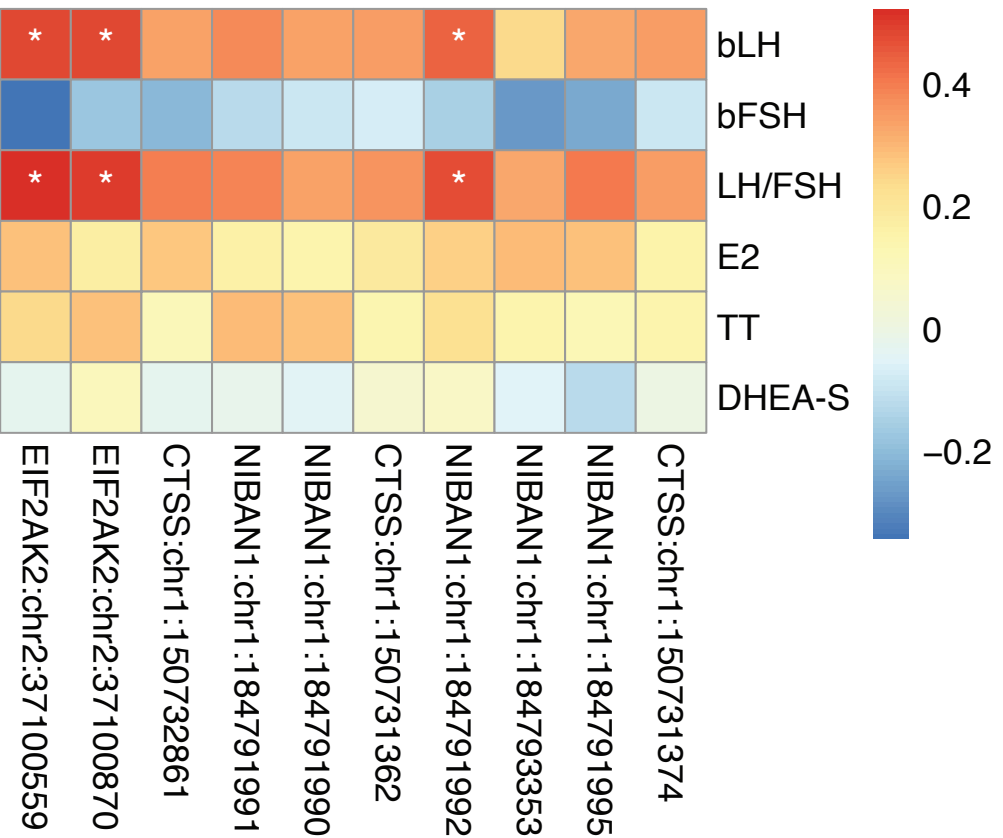

Supplement: Supplementary file 6 — Additional file 6: Fig S5. Correlation analysis between the ten hub RNA editing events and clinical hormone indices in PCOS patients. [file 12916_2024_3434_MOESM6_ESM.pdf]

(A)

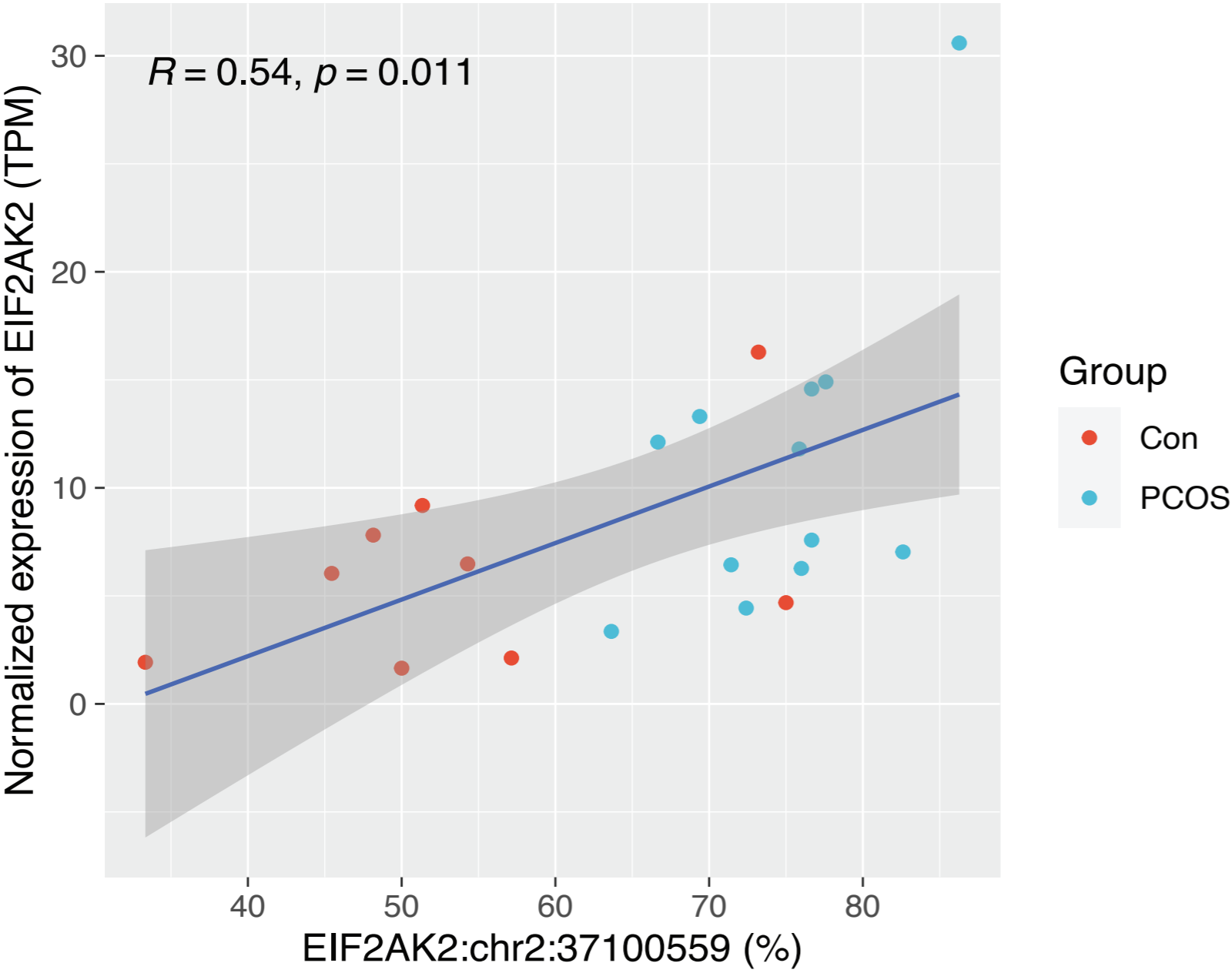

(C)

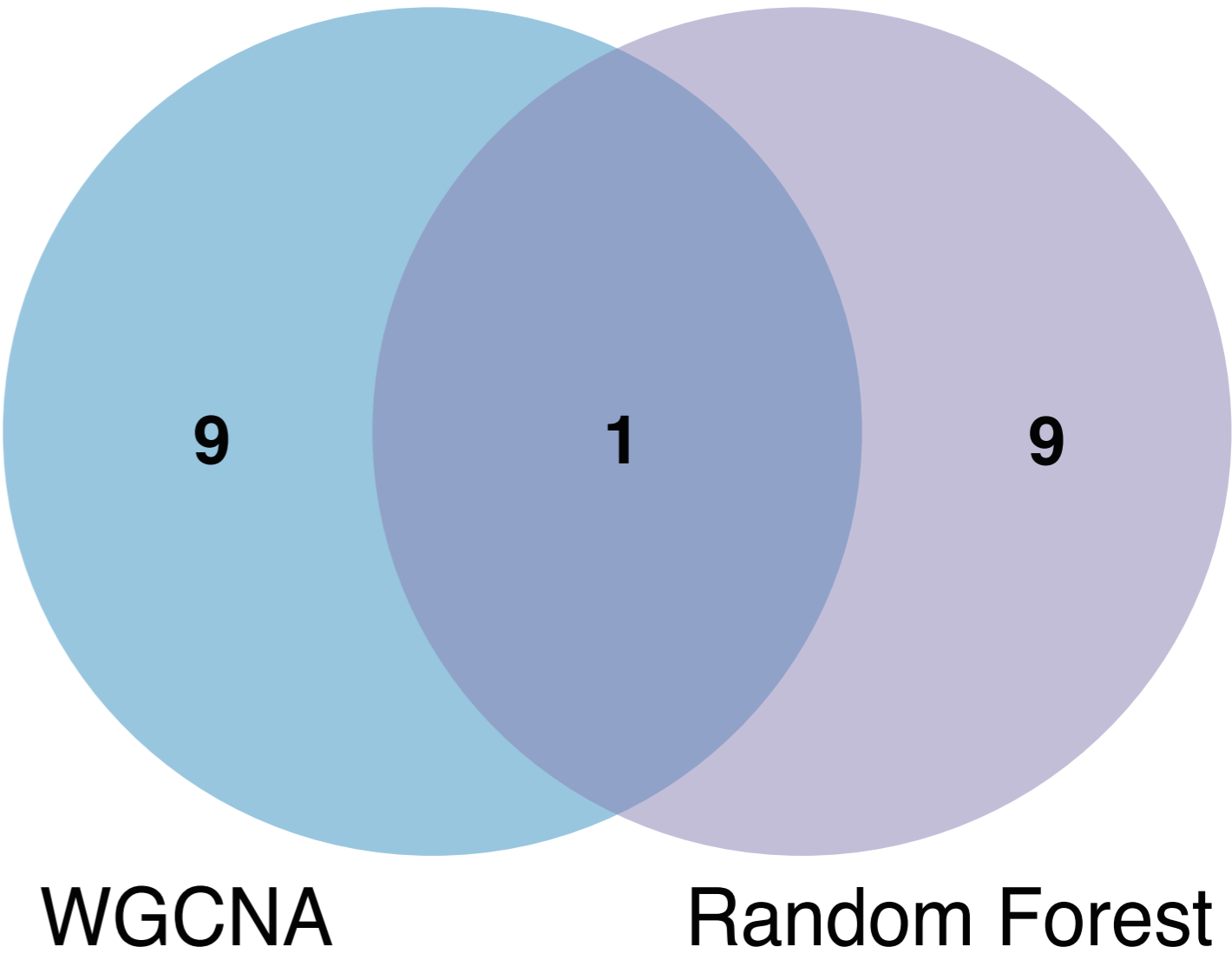

(B)

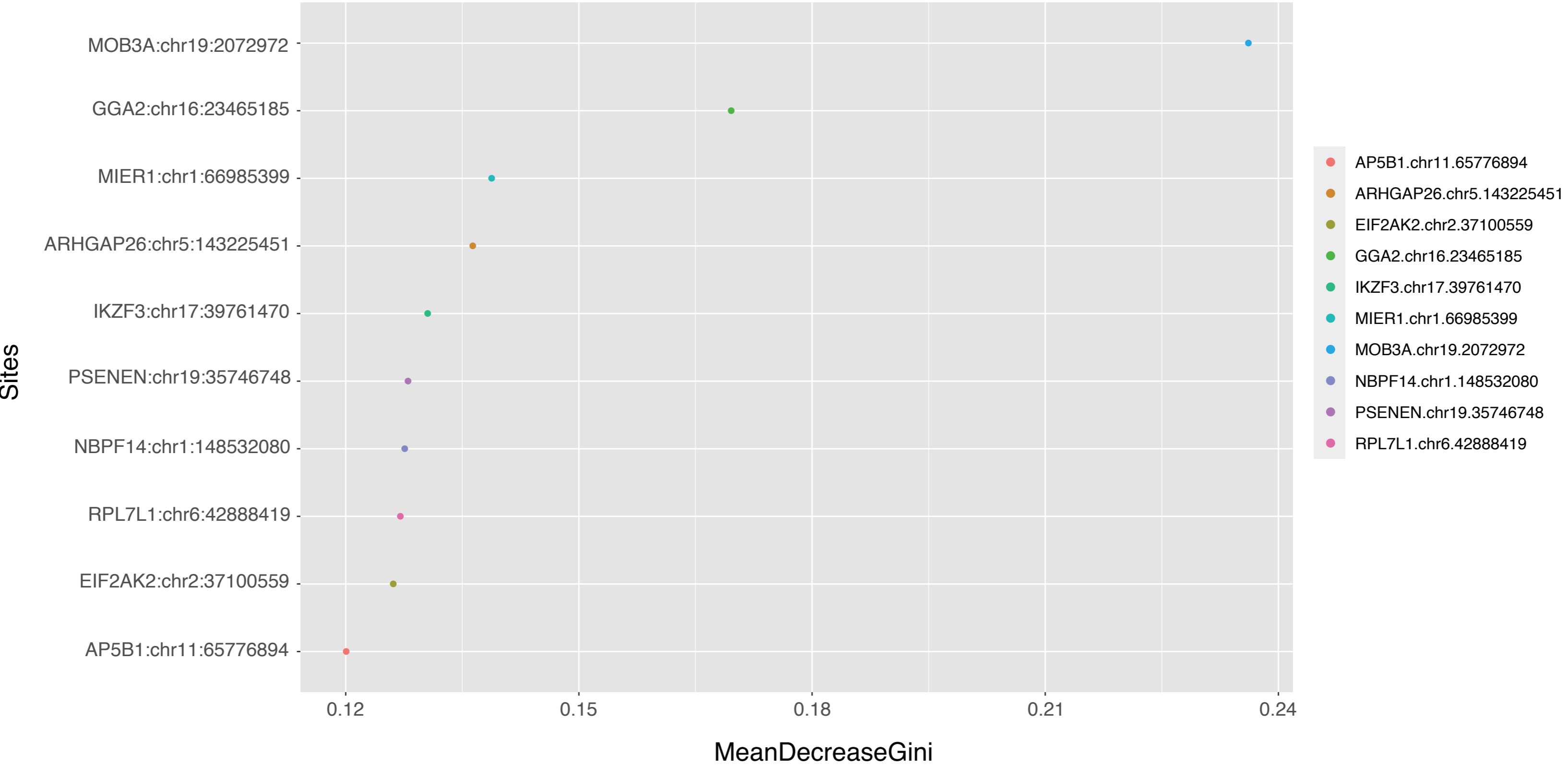

Supplement: Supplementary file 7 — Additional file 7: Fig S6. (A) Analysis of cis-regulatory effects of the editing level of EIF2AK2:chr2:37,100,559 on EIF2AK2 expression. (B) Random Forest analysis identifying the ten hub RNA editing events based on their impact on PCOS phenotype prediction. (C) Venn diagram showing the overlap of significant editing events identified by Random Forest analysis and WGCNA. [file 12916_2024_3434_MOESM7_ESM.pdf]

(A)

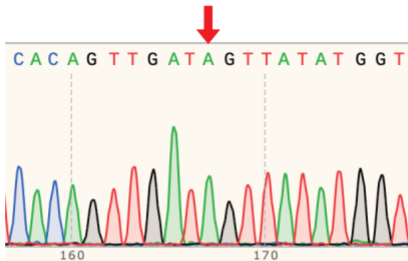

(B)

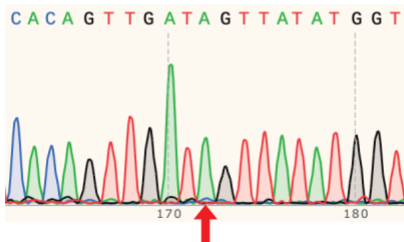

Supplement: Supplementary file 8 — Additional file 8: Fig S7. Verification of RNA editing event. 10 bp upstream and 10 bp downstream surrounding the editing site (EIF2AK2:chr2:37,100,559) in control (A) and PCOS (B) blood samples. [file 12916_2024_3434_MOESM8_ESM.pdf]

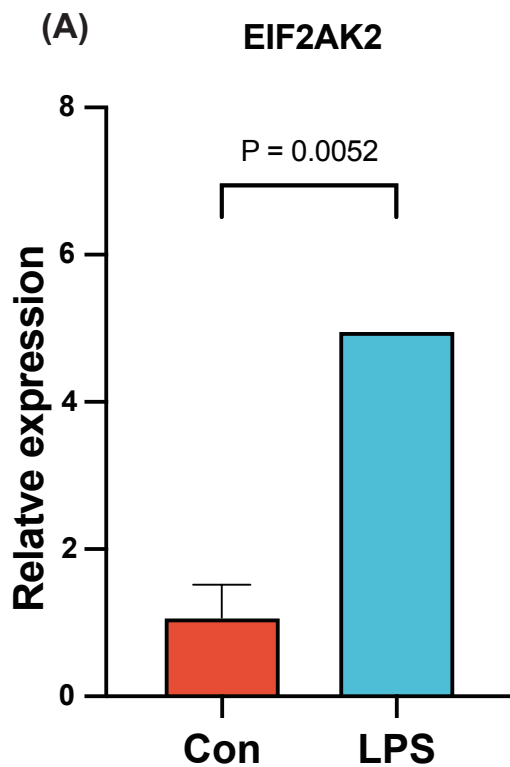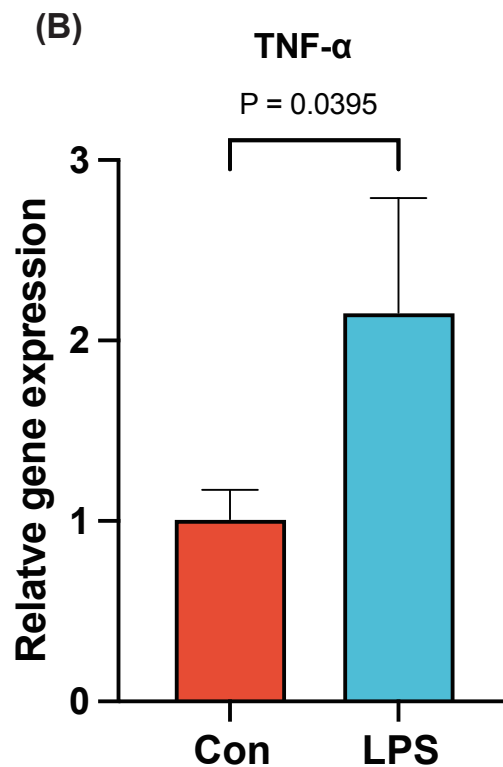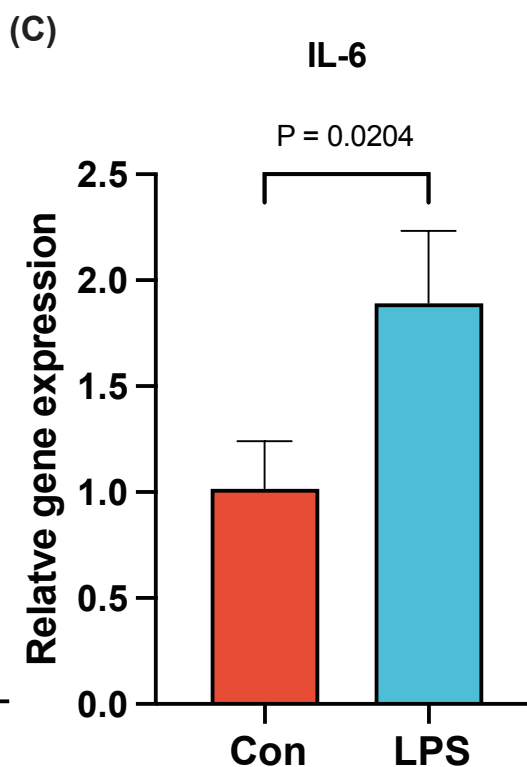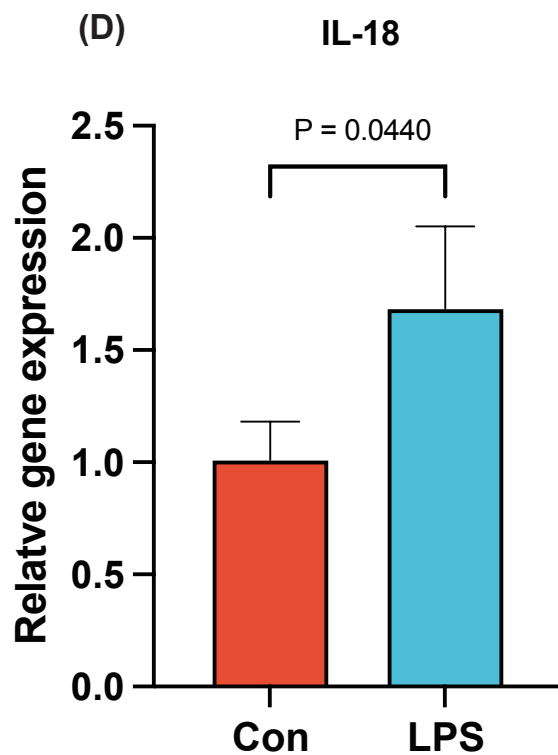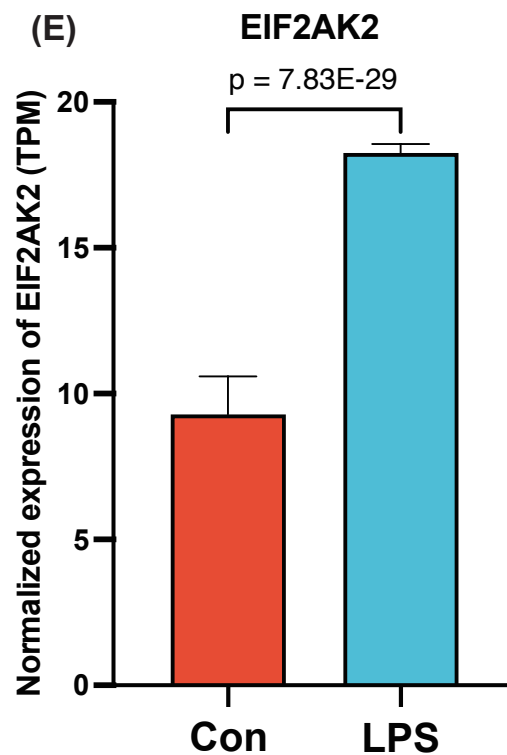

Supplement: Supplementary file 9 — Additional file 9: Fig S8. (A) Expression level of EIF2AK2 in THP-1 cells 24 h post-stimulation with 1 µg/mL lipopolysaccharide (LPS). (B-D) Expression levels of inflammatory cytokines in response to LPS stimulation. (E) EIF2AK2 expression in THP-1 cells from dataset PRJNA993124 after LPS stimulation. [file 12916_2024_3434_MOESM9_ESM.pdf]

(A) ALDH6A1:chr14:74058840 (B) ERCC2:chr19:45350155 (C) MDM2:chr12:68843263 (D) AC008764.4:chr19:16634547

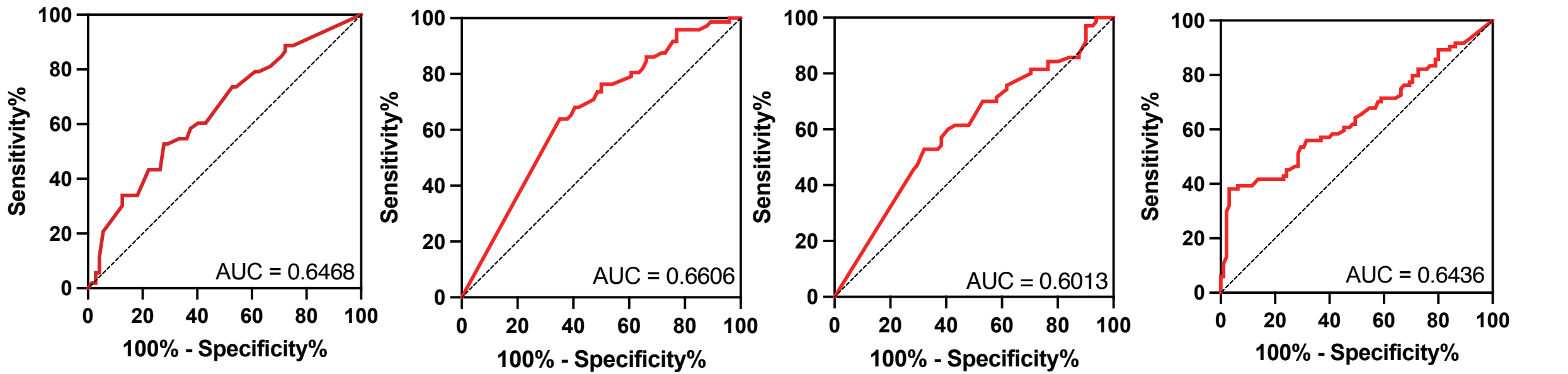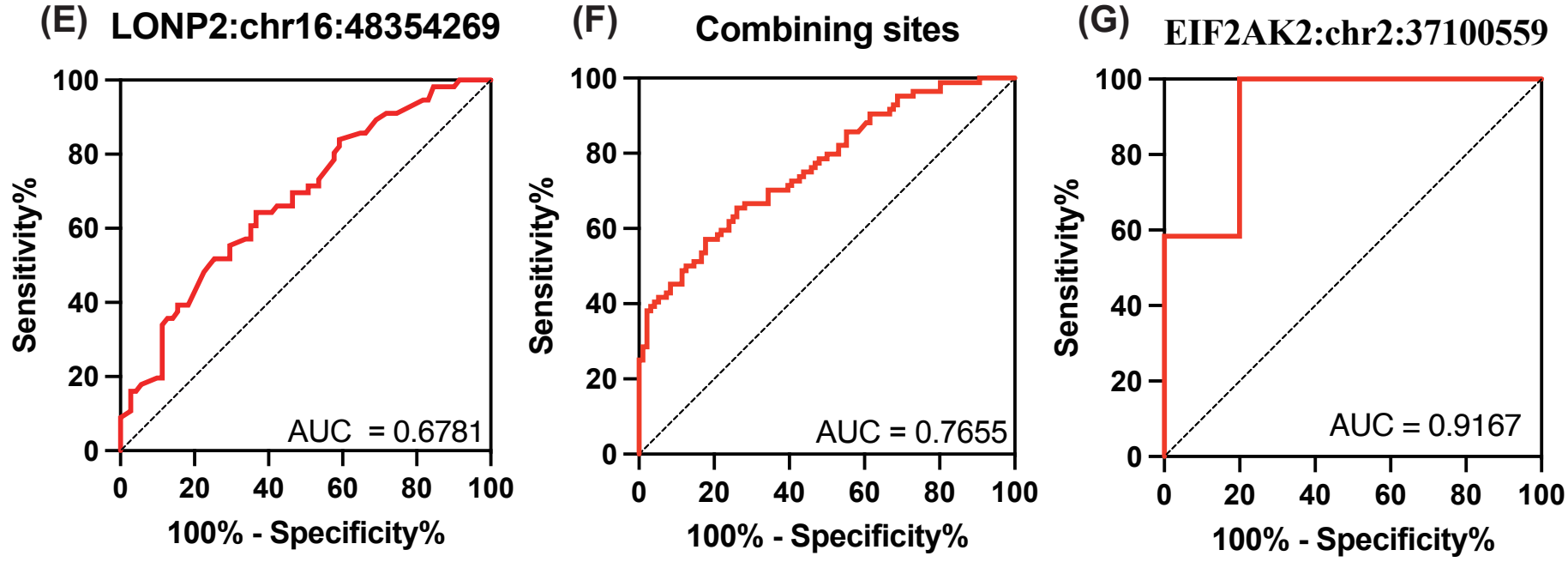

Supplement: Supplementary file 10 — Additional file 10: Fig S9. ROC curves giving the diagnostic value of editing events in PCOS. (A-E) The AUC value of RNA editing levels of combined ERCC2:chr19:45,350,155, ALDH6A1:chr14:74,058,840,LONP2:chr16:48,354,269, MDM2:chr12:68,843,263 and AC008764.4:chr19:16,634,547 in dataset PRJNA719824, respectively. (F) The AUC value of the five combined editing events in dataset PRJNA719824. (G) The AUC value of the classifier generated by RNA editing level of EIF2AK2:chr2:37,100,559 in peripheral blood samples of PCOS cohort. [file 12916_2024_3434_MOESM10_ESM.pdf]

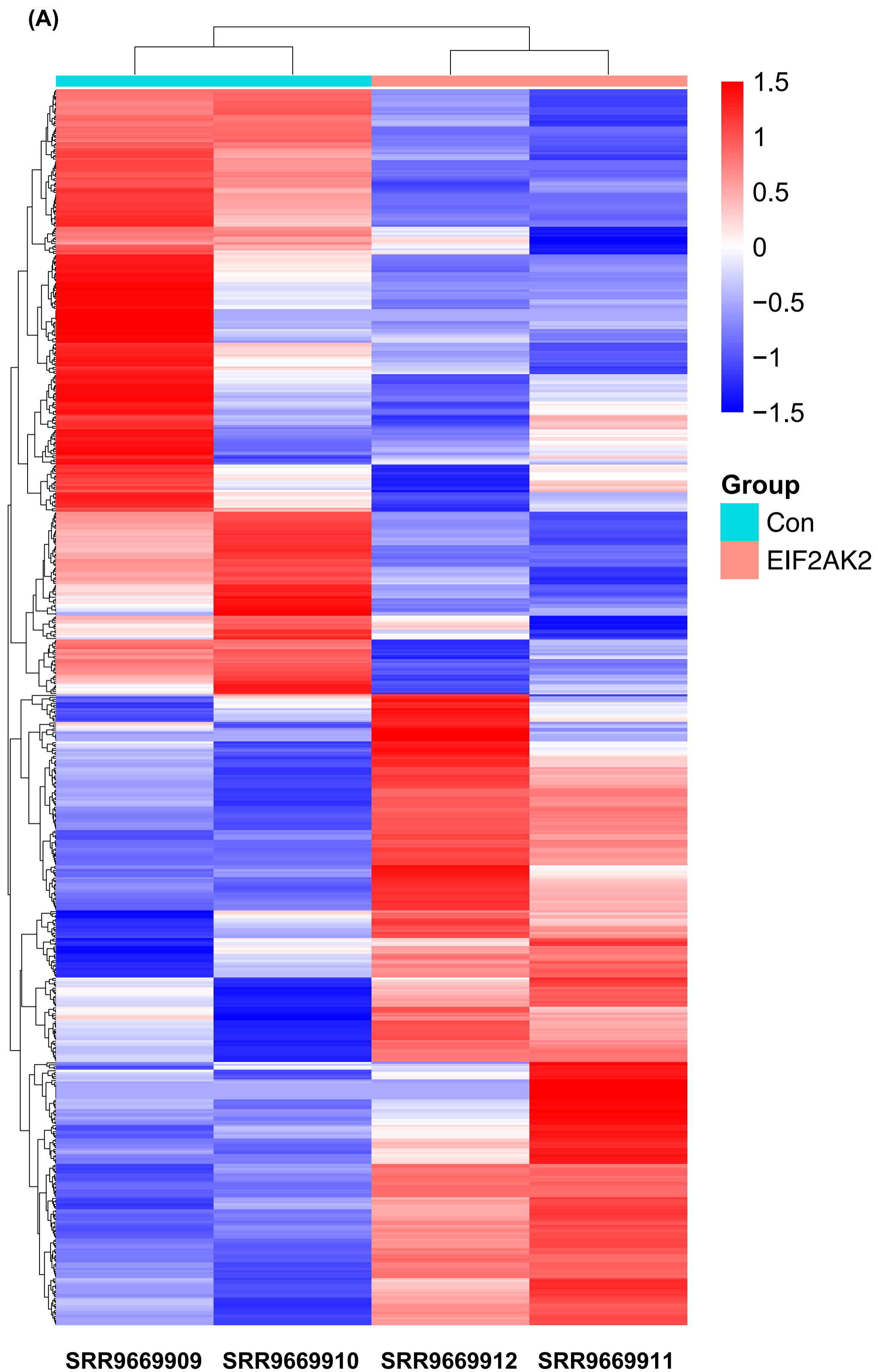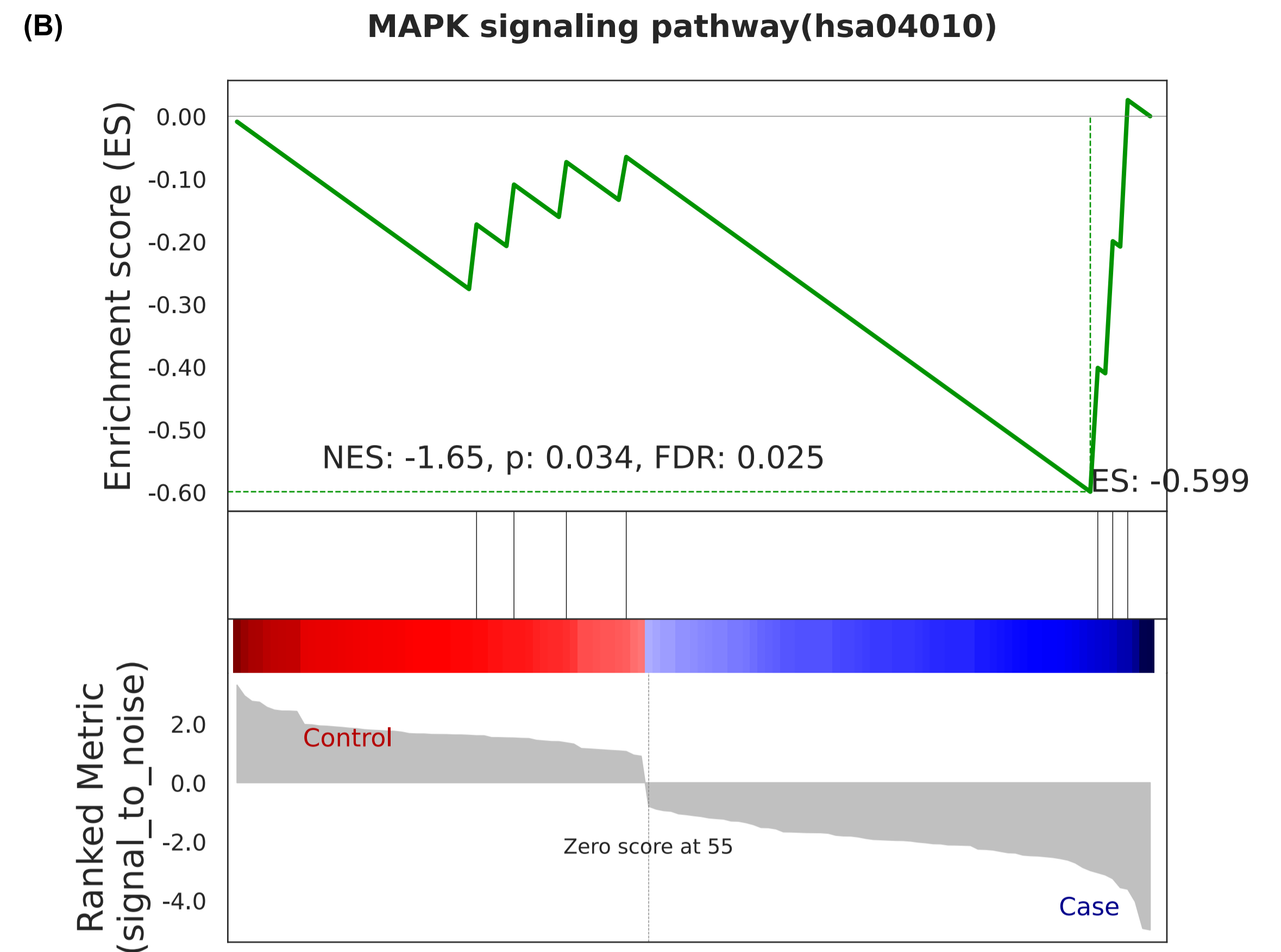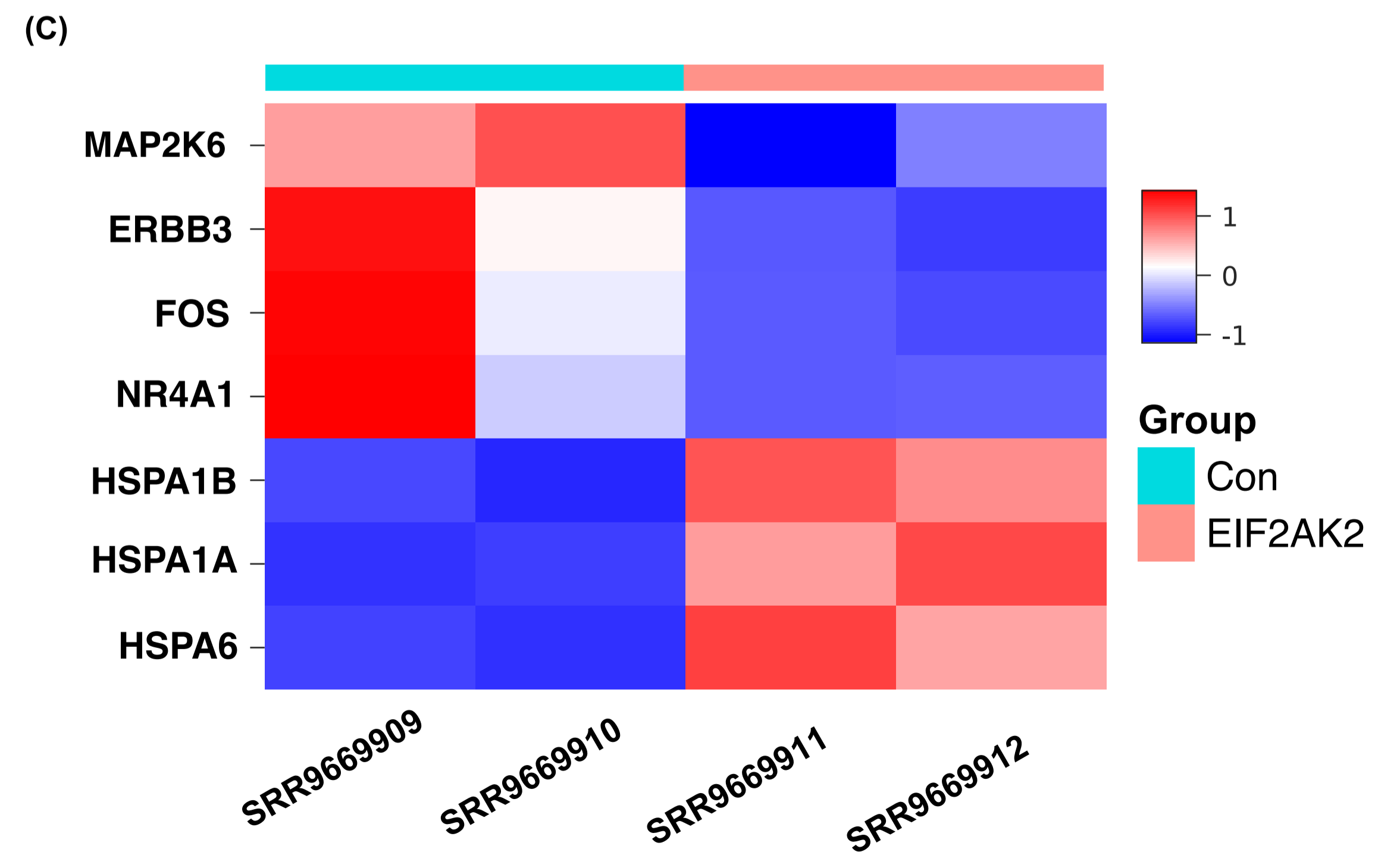

Supplement: Supplementary file 11 — Additional file 11: Fig S10. (A) Heatmap of 123 differentially expressed genes based on the criteria of FDR ≤ 0.05, |logFC|≥ 1 in dataset PRJNA554006. (B) Analysis of the MAPK signaling pathway via gene set enrichment analysis (GSEA). (C) Expression heatmap of seven MAPK-related genes in dataset PRJNA554006. [file 12916_2024_3434_MOESM11_ESM.pdf]
