# Supplementary material for: Dysregulated RNA editing of EIF2AK2 in polycystic ovary syndrome: clinical relevance and functional implications
Source: BMC Med. 2024 Jun 10;22:229. doi: 10.1186/s12916-024-03434-8 (PMC11163819; doi:10.1186/s12916-024-03434-8)
Supplement: Supplementary file 4 — Additional file 4: Fig S3. Prediction of RNA-binding proteins (RBPs) that potentially interact with differential editing events identified in at least three separate datasets. [file 12916_2024_3434_MOESM4_ESM.pdf]

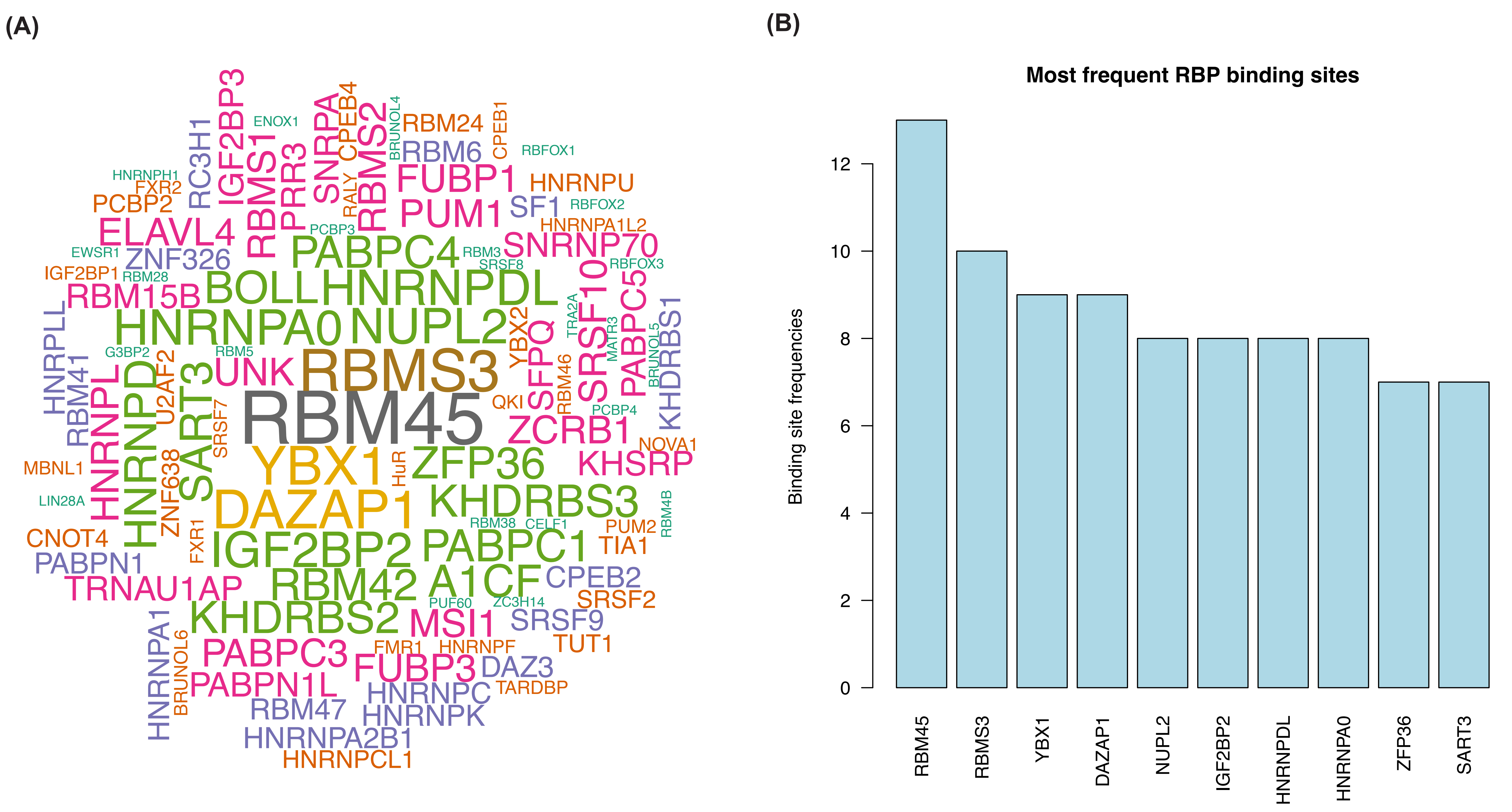
(A)
RBM45
RBMS3
YBX1
DAZAP1
NUPL2
IGF2BP2
HNRNPDL
HNRNPA0
ZFP36
SART3
KHDRBS3
KHDRBS2
RBM42
A1CF
PABPC1
PABPC4
BOLL
HNRNPD
HNRNPL
ELAVL4
PUM1
FUBP1
SNRNP70
SRSF10
PABPC5
ZCRB1
KHSRP
KHDRBS1
SFPQ
YBX2
UNK
RBM15B
ZNF326
IGF2BP3
RBMS1
RBMS2
PRR3
SNRPA
CPEB4
RALY
RC3H1
RBM24
RBM6
CPEB1
HNRNPU
SF1
RBFOX1
RBFOX2
RBFOX3
HNRNPA1L2
HNRNPH1
FXR2
PCBP2
PCBP3
ENOX1
BRUNOL4
EWSR1
IGF2BP1
RBM28
RBM3
SRSF8
HNRPLL
RBM41
G3BP2
U2AF2
RBM5
SRSF7
ZNF638
HuR
QKI
RBM46
TRA2A
MATR3
BRUNOL5
PCBP4
NOVA1
MBNL1
LIN28A
CNOT4
PABPN1
TRNAU1AP
FXR1
RBM38
CELF1
PUM2
TIA1
RBM4B
CPEB2
SRSF2
PUF60
ZC3H14
MSI1
SRSF9
TUT1
HNRNPA1
BRUNOL6
PABPC3
PABPN1L
FMR1
HNRNPF
FUBP3
DAZ3
TARDBP
HNRNPC
HNRNPK
RBM47
HNRNPA2B1
HNRNPCL1
(B)
Most frequent RBP binding sites
Binding site frequencies
0
2
4
6
8
10
12
RBM45
RBMS3
YBX1
DAZAP1
NUPL2
IGF2BP2
HNRNPDL
HNRNPA0
ZFP36
SART3
